# Supplementary material for: Doubling multiplexed imaging capability via spatial expression pattern-guided protein pairing and computational unmixing
Source: Commun Biol. 2025 Jun 14;8:928. doi: 10.1038/s42003-025-08357-5 (PMC12167378; doi:10.1038/s42003-025-08357-5)
Supplement: Supplementary file 1 — Supplementary Information [file 42003_2025_8357_MOESM1_ESM.pdf]

SUPPLEMENTARY FIGURES

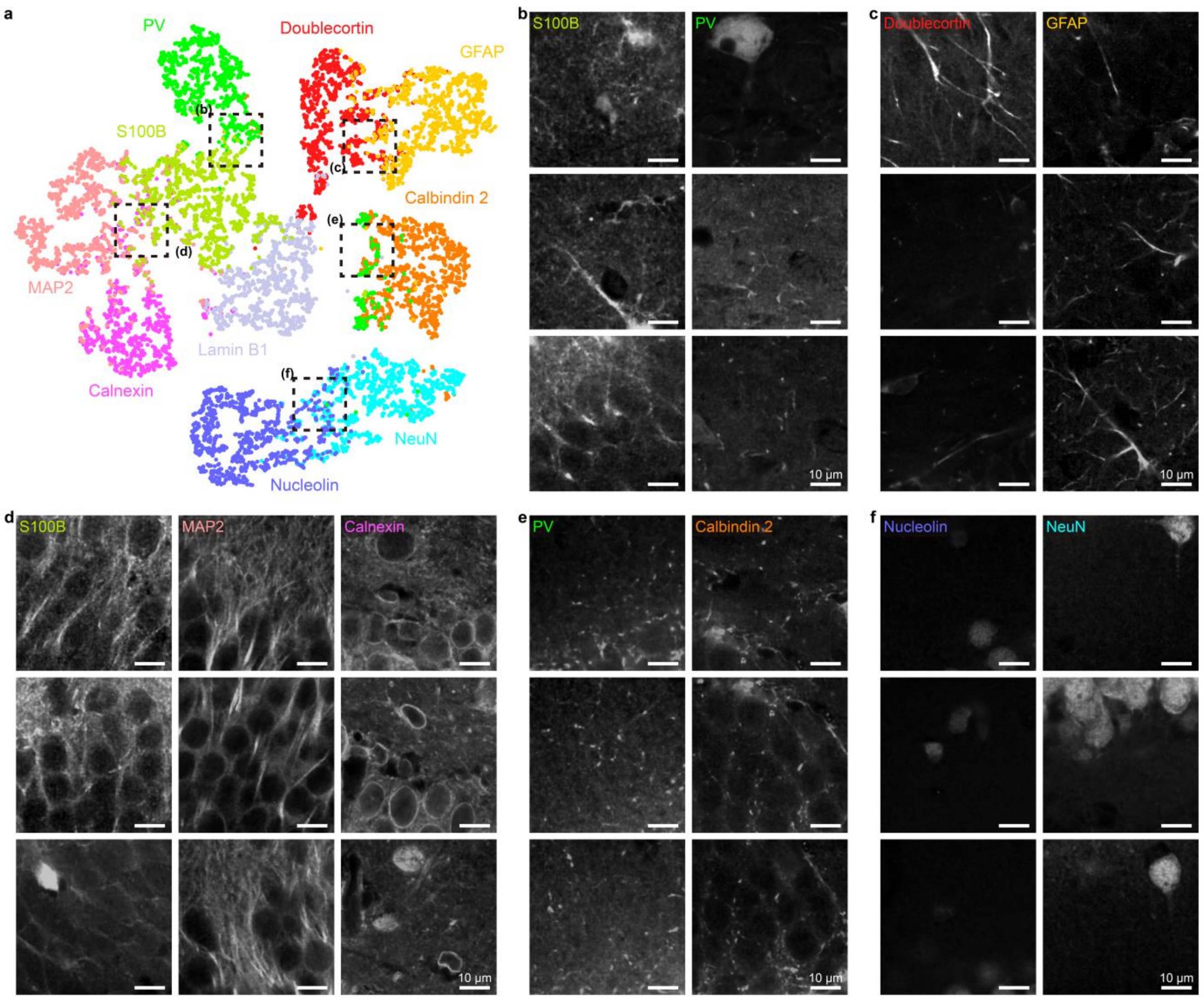

Supplementary Figure 1. Visualization of protein images that are closely located in the feature domain. a, The t-SNE plot of extracted feature vectors from each protein image patch and boxed regions showing protein pairs that are closely located in the feature domain due to their similar spatial expression patterns. **b-f**, Image patches corresponding to the boxed region in **a**. **b,** S100B and PV; **c**, doublecortin and GFAP; **d**, S100B, MAP2, and calnexin; **e**, PV and calbindin 2; **f**, nucleolin and NeuN. Scale bar = 10 μm in **b-f**.

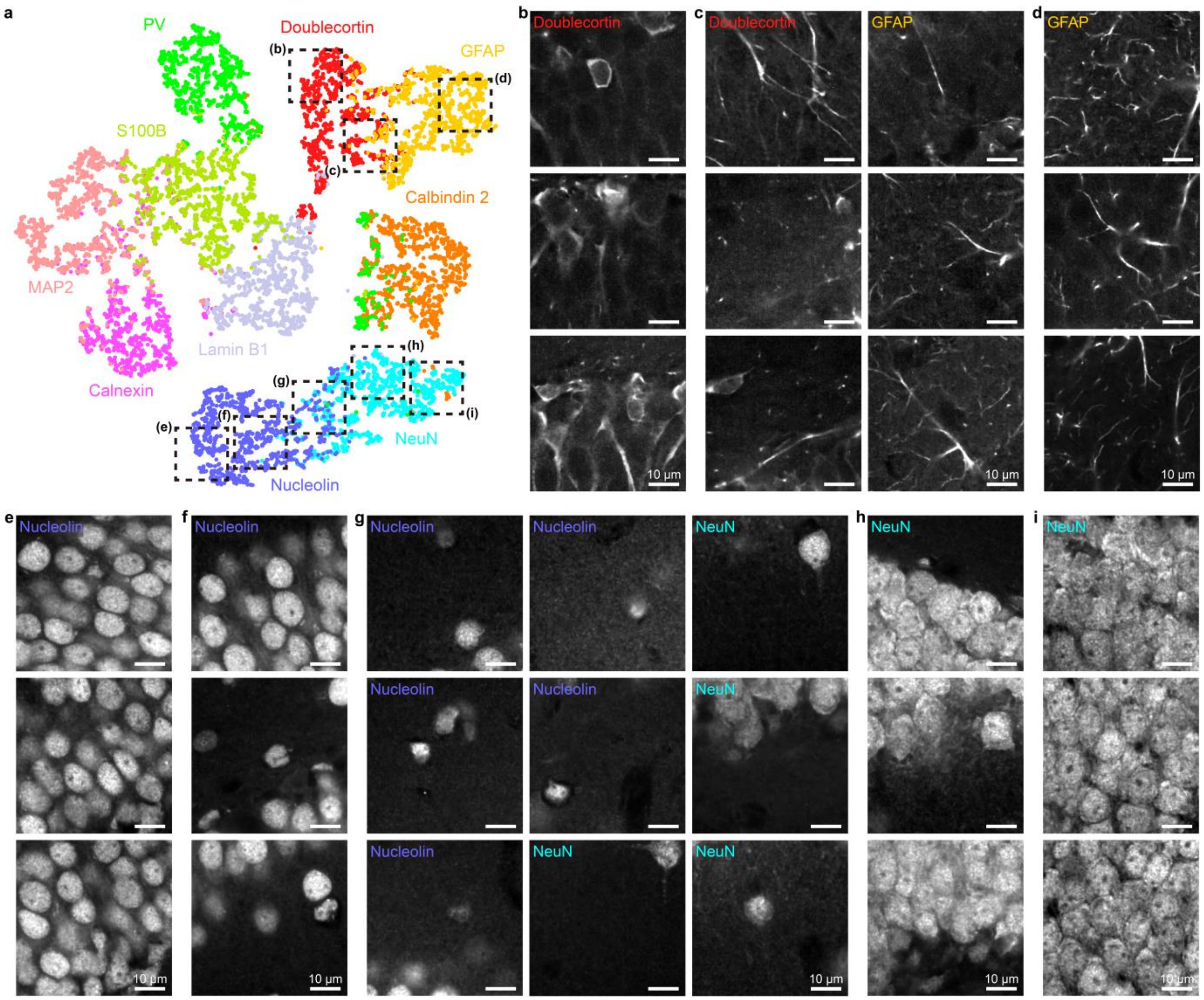

Supplementary Figure 2. Visualization of protein images that are closely located in the feature domain—non-overlapping region to overlapping region. a, The t-SNE plot of extracted feature vectors from each protein image patch and boxed regions showing protein pairs that are closely located in the feature domain due to their similar spatial expression patterns. **b-i**, Image patches corresponding to the boxed region in **a**. **b-d**, Doublecortin and GFAP. **b**, Doublecortin in the non-overlapping region; **c**, Doublecortin and GFAP in the overlapping region; **d**, GFAP in the non-overlapping region. **e-i**, Nucleolin and NeuN. **e-f**, Nucleolin in the non-overlapping region; **g**, Nucleolin and NeuN in the overlapping region; **h-i**, NeuN in the non-overlapping region. Scale bar = 10 μm in **b-i**.

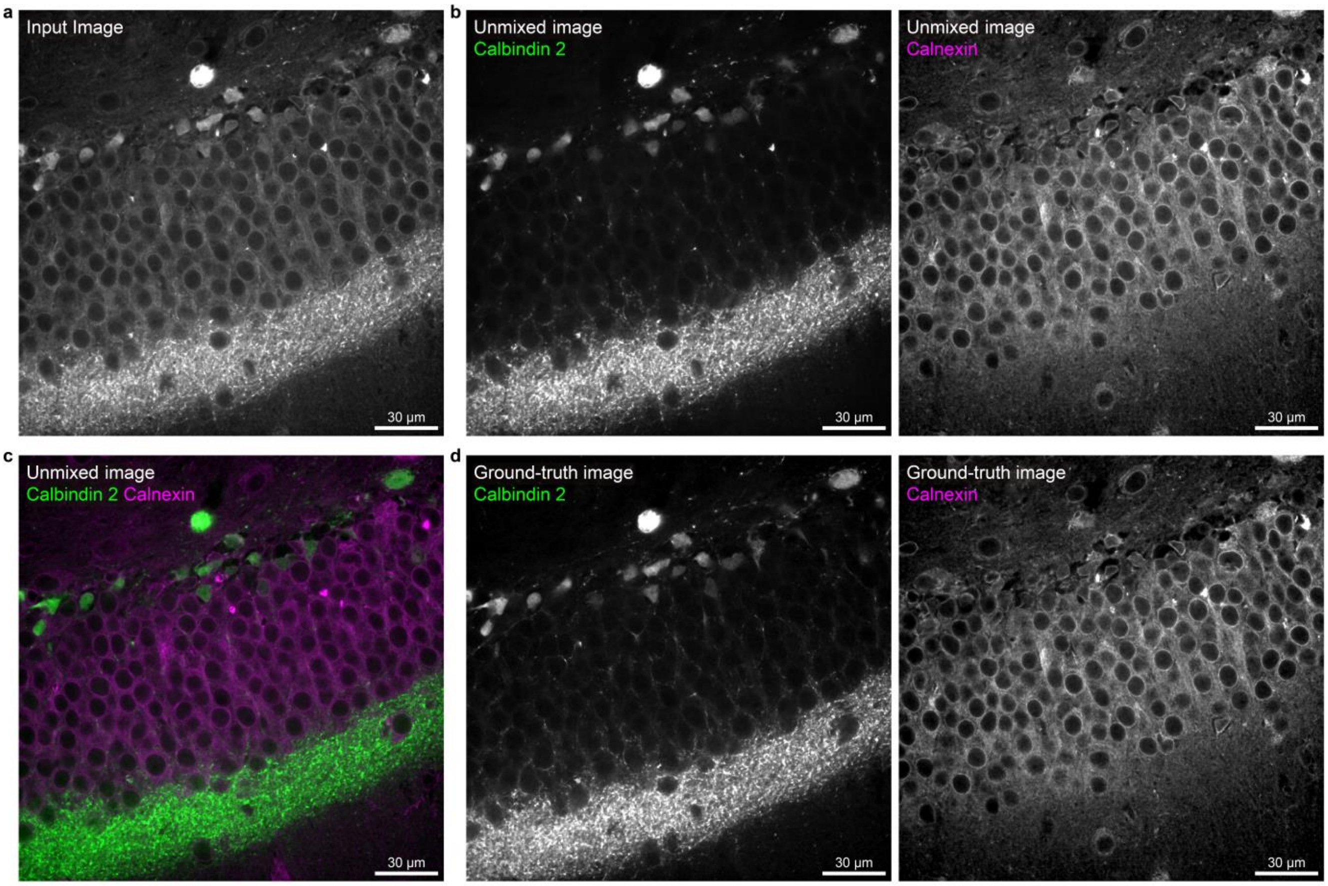

Supplementary Figure 3. Visualization of the unmixing results: calbindin2 and calnexin. a, Input gray scale image. **b**, Unmixed individual channel images for each protein. **c**, Unmixed image represented as channel-wise merge using a green-and-magenta color scheme, with calbindin 2 in green and calnexin in magenta. **d**, Ground-truth image of each protein. Scale bar = 30 µm in **a-d**.

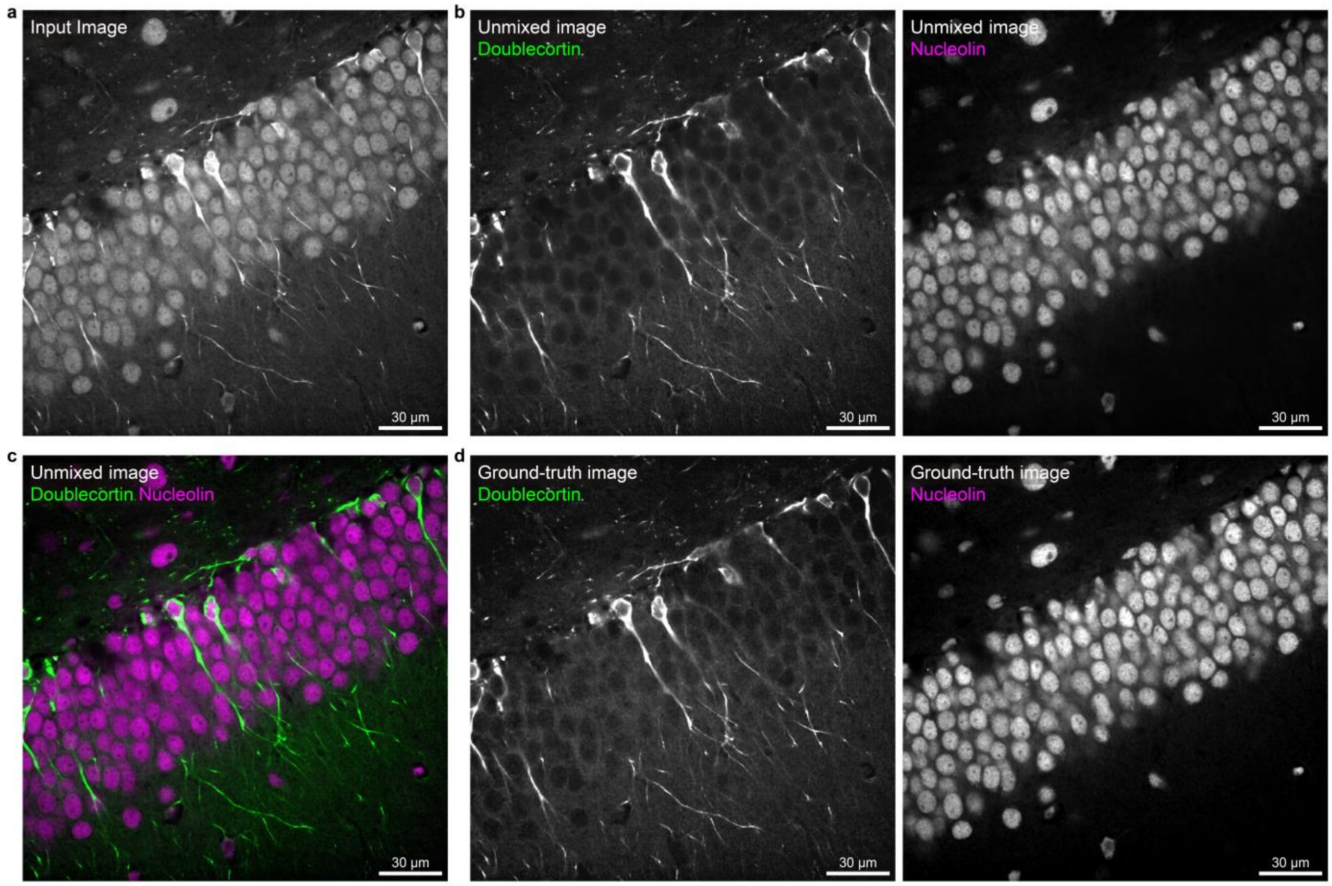

Supplementary Figure 4. Visualization of the unmixing results: doublecortin and nucleolin. a, Input gray scale image. **b**, Unmixed individual channel images for each protein. **c**, Unmixed image represented as channel-wise merge using a green-and-magenta color scheme, with doublecortin in green and nucleolin in magenta. **d**, Ground-truth image of each protein. Scale bar = 30 μm in **a-d**.

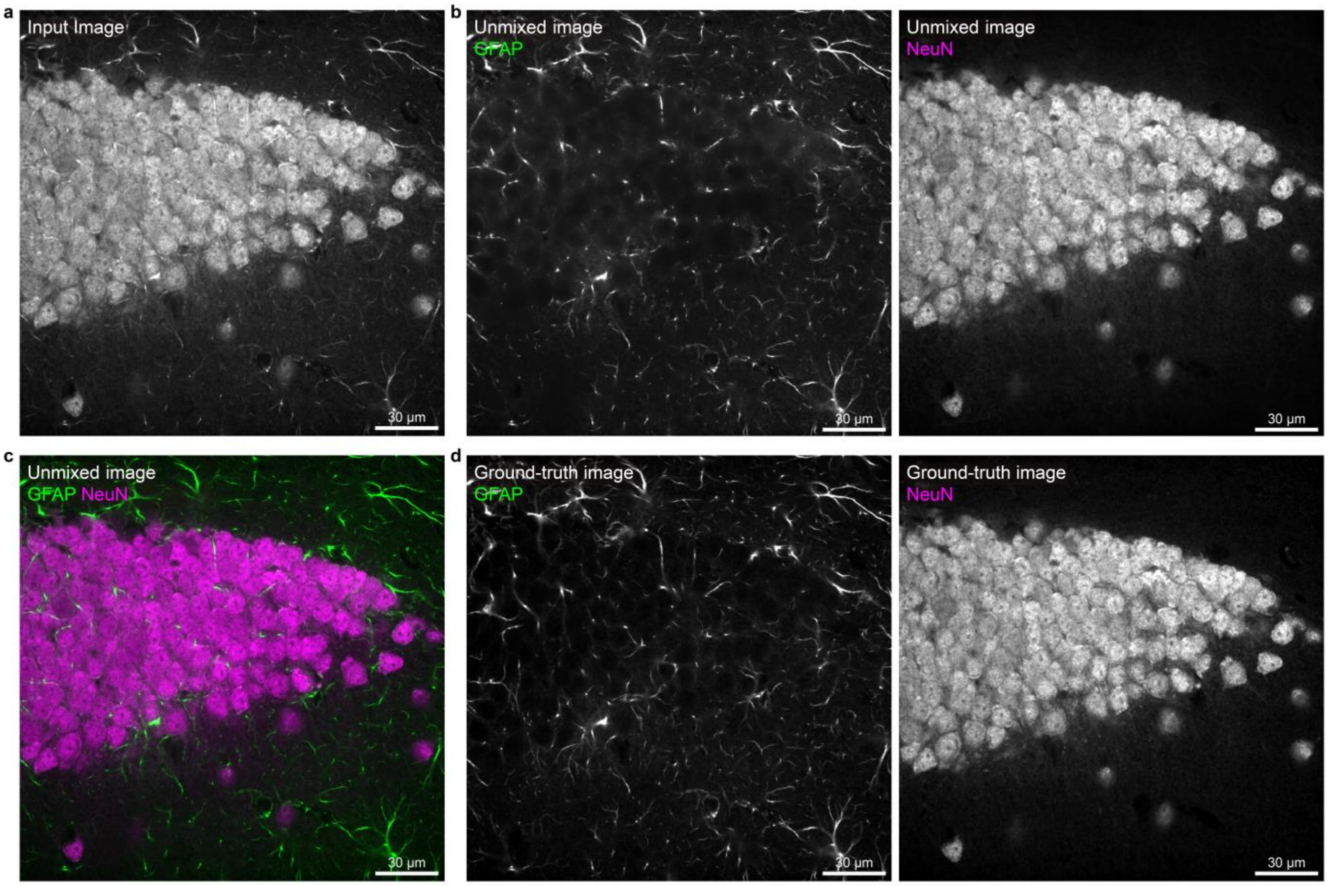

Supplementary Figure 5. Visualization of the unmixing results: GFAP and NeuN. a, Input gray scale image. **b**, Unmixed individual channel images for each protein. **c**, Unmixed image represented as channel-wise merge using a green-and-magenta color scheme, with GFAP in green and NeuN in magenta. **d**, Ground-truth image of each protein. Scale bar = 30 µm in **a-d**.

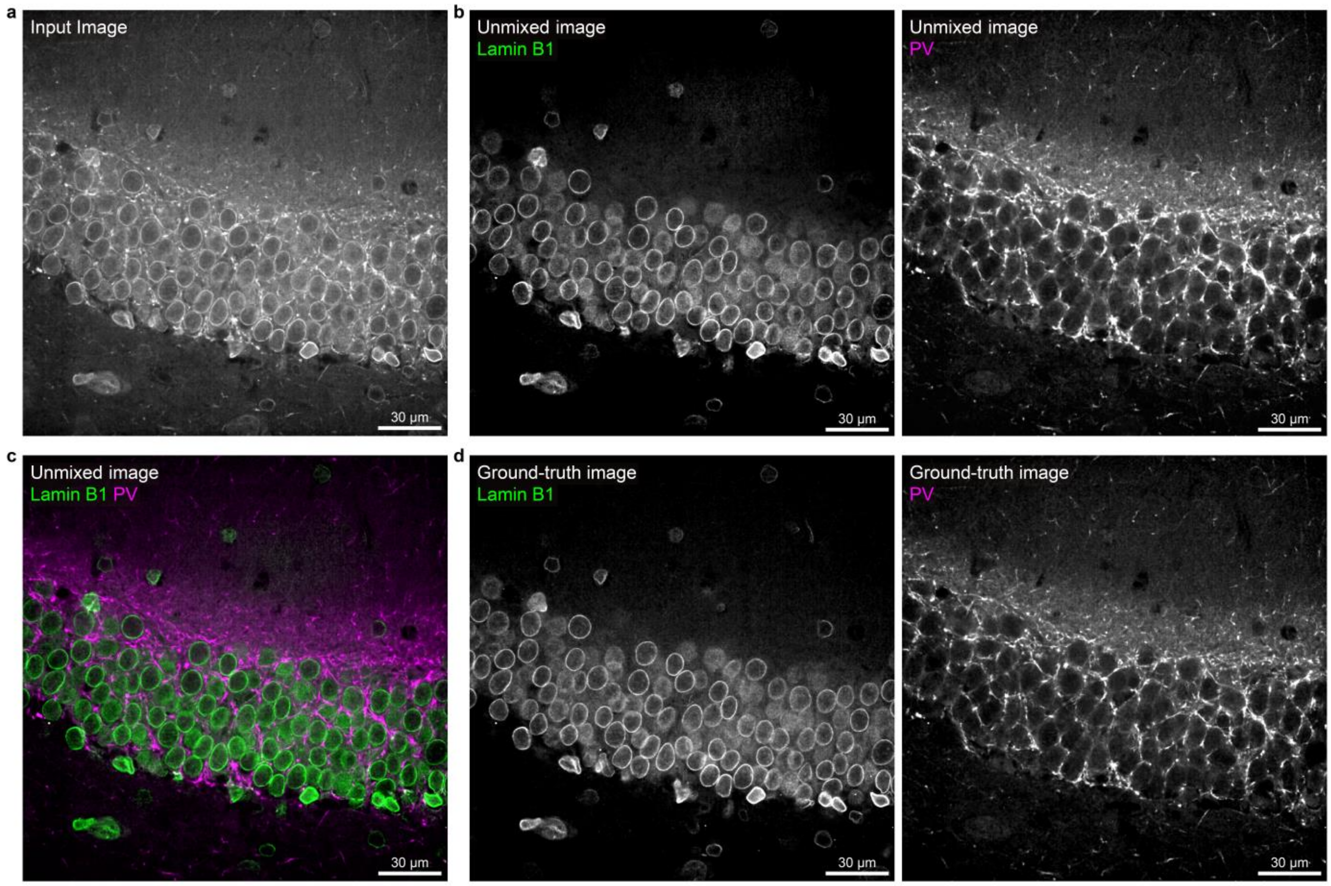

Supplementary Figure 6. Visualization of the unmixing results: lamin B1 and PV. a, Input gray scale image. **b**, Unmixed individual channel images for each protein. **c**, Unmixed image represented as channel-wise merge using a green-and-magenta color scheme, with lamin B1 in green and PV in magenta. **d**, Ground-truth image of each protein. Scale bar = 30 µm in **a-d**.

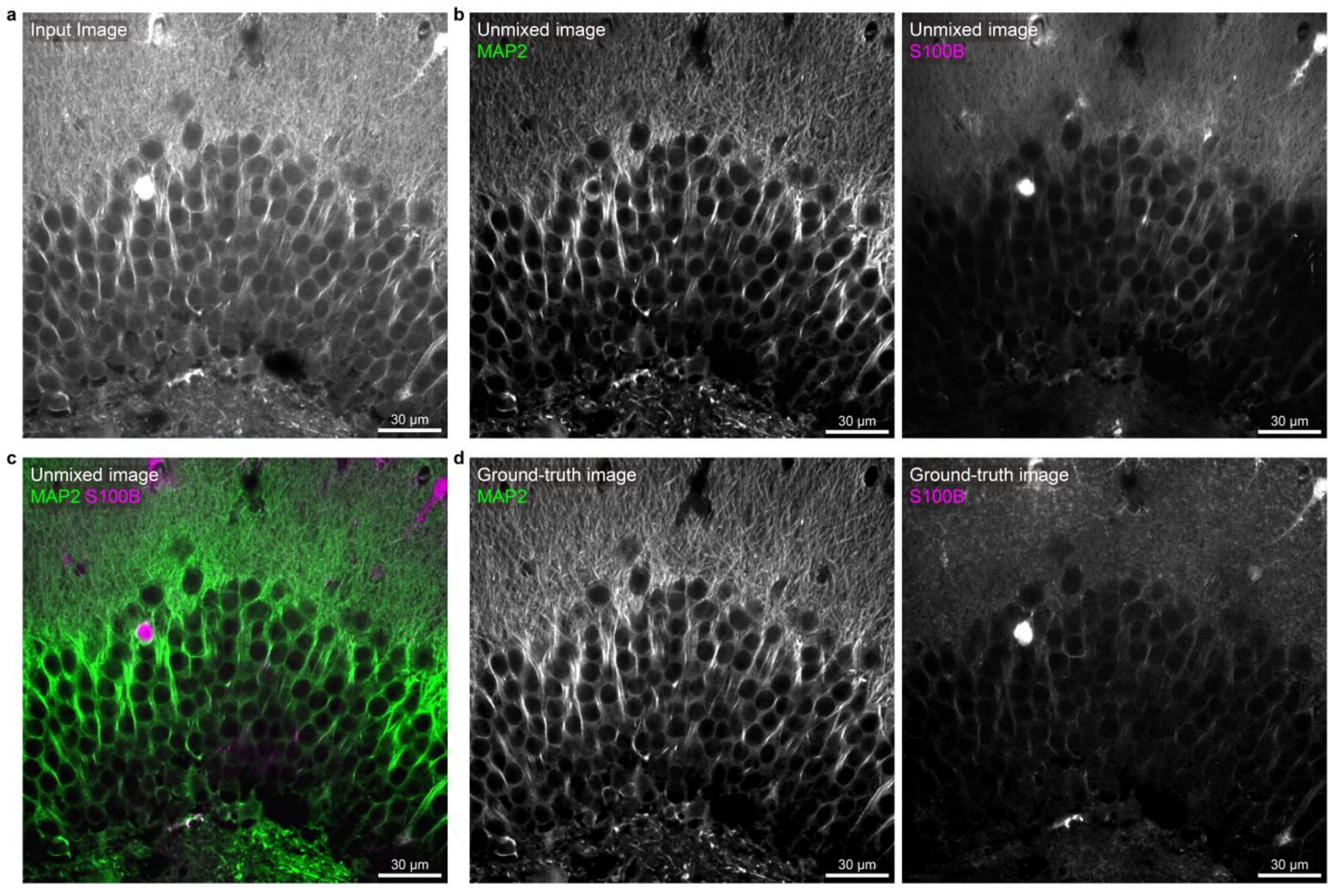

Supplementary Figure 7. Visualization of the unmixing results: MAP2 and S100B. a, Input gray scale image. **b**, Unmixed individual channel images for each protein. **c**, Unmixed image represented as channel-wise merge using a green-and-magenta color scheme, with MAP2 in green and S100B in magenta. **d**, Ground-truth image of each protein. Scale bar = 30 µm in **a-d**.

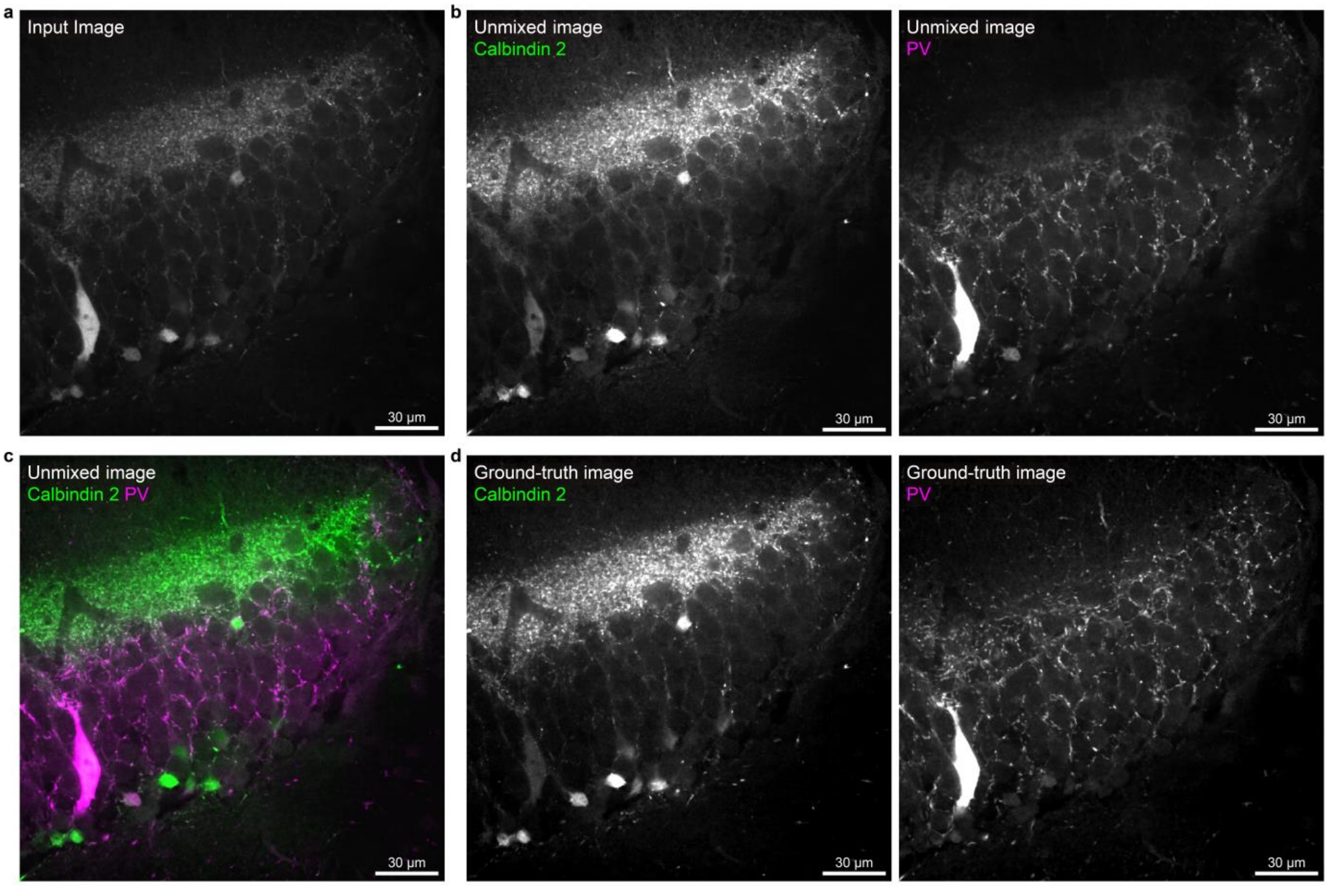

Supplementary Figure 8. Visualization of the unmixing results: calbindin 2 and PV. a, Input gray scale image. **b**, Unmixed individual channel images for each protein. **c**, Unmixed image represented as channel-wise merge using a green-and-magenta color scheme, with calbindin 2 in green and PV in magenta. **d**, Ground-truth image of each protein. Scale bar = 30 µm in **a-d**.

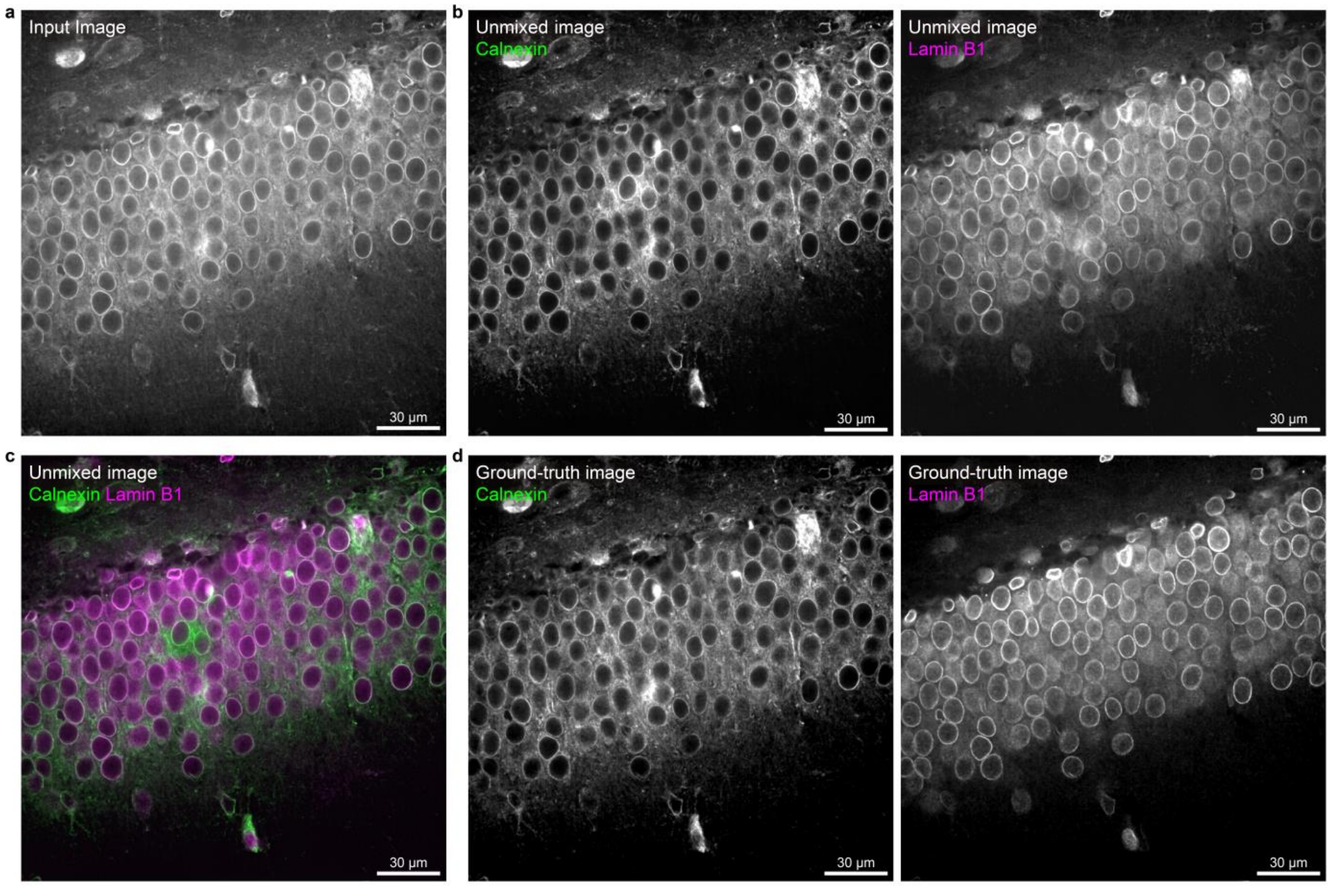

Supplementary Figure 9. Visualization of the unmixing results: calnexin and lamin B1. a, Input gray scale image. **b**, Unmixed individual channel images for each protein. **c**, Unmixed image represented as channel-wise merge using a green-and-magenta color scheme, with calnexin in green and lamin B1 in magenta. **d**, Ground-truth image of each protein. Scale bar = 30 µm in **a-d**.

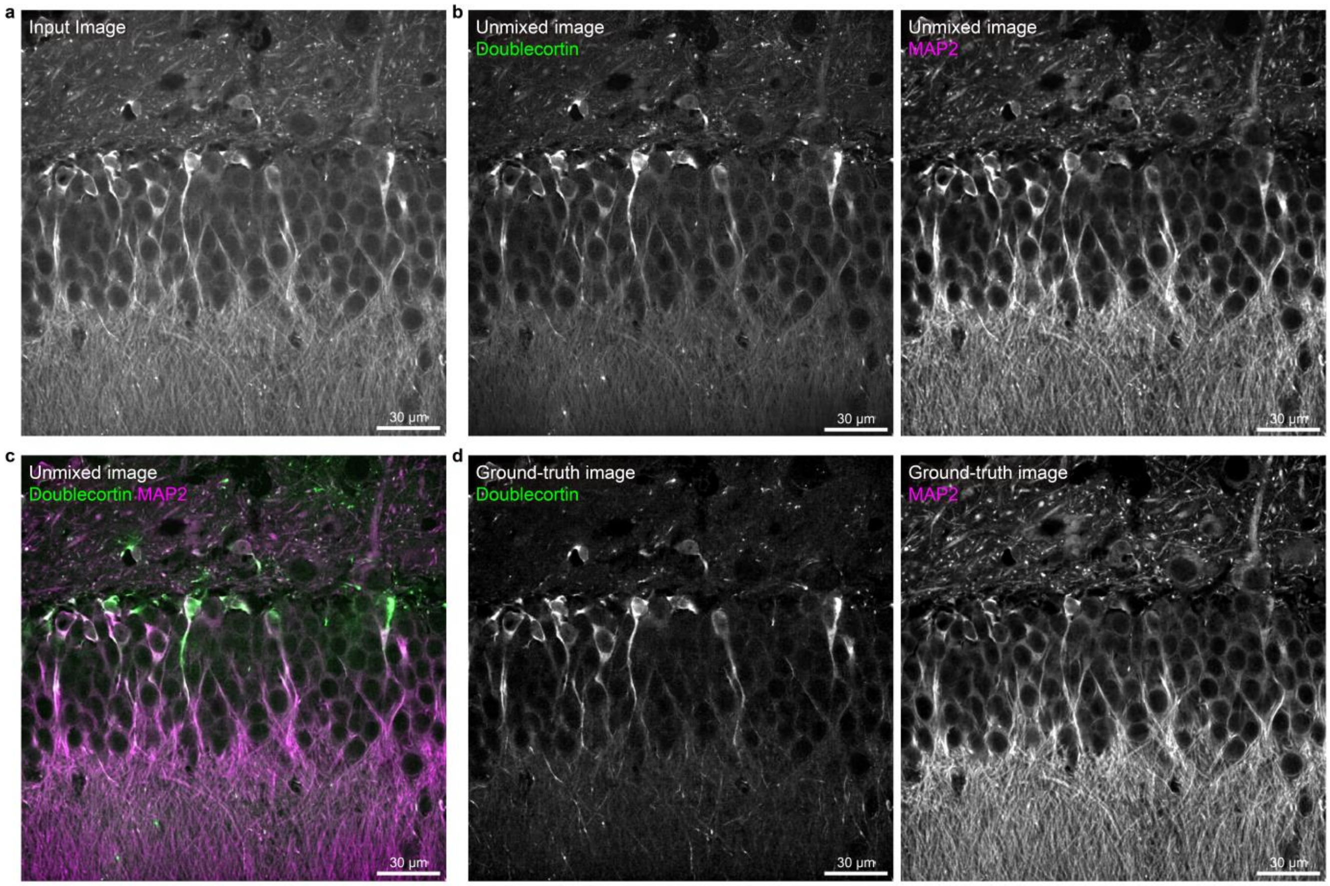

Supplementary Figure 10. Visualization of the unmixing results: doublecortin and MAP2. a, Input gray scale image. **b**, Unmixed individual channel images for each protein. **c**, Unmixed image represented as channel-wise merge using a green-and-magenta color scheme, with doublecortin in green and MAP2 in magenta. **d**, Ground-truth image of each protein. Scale bar = 30 µm in **a-d**.

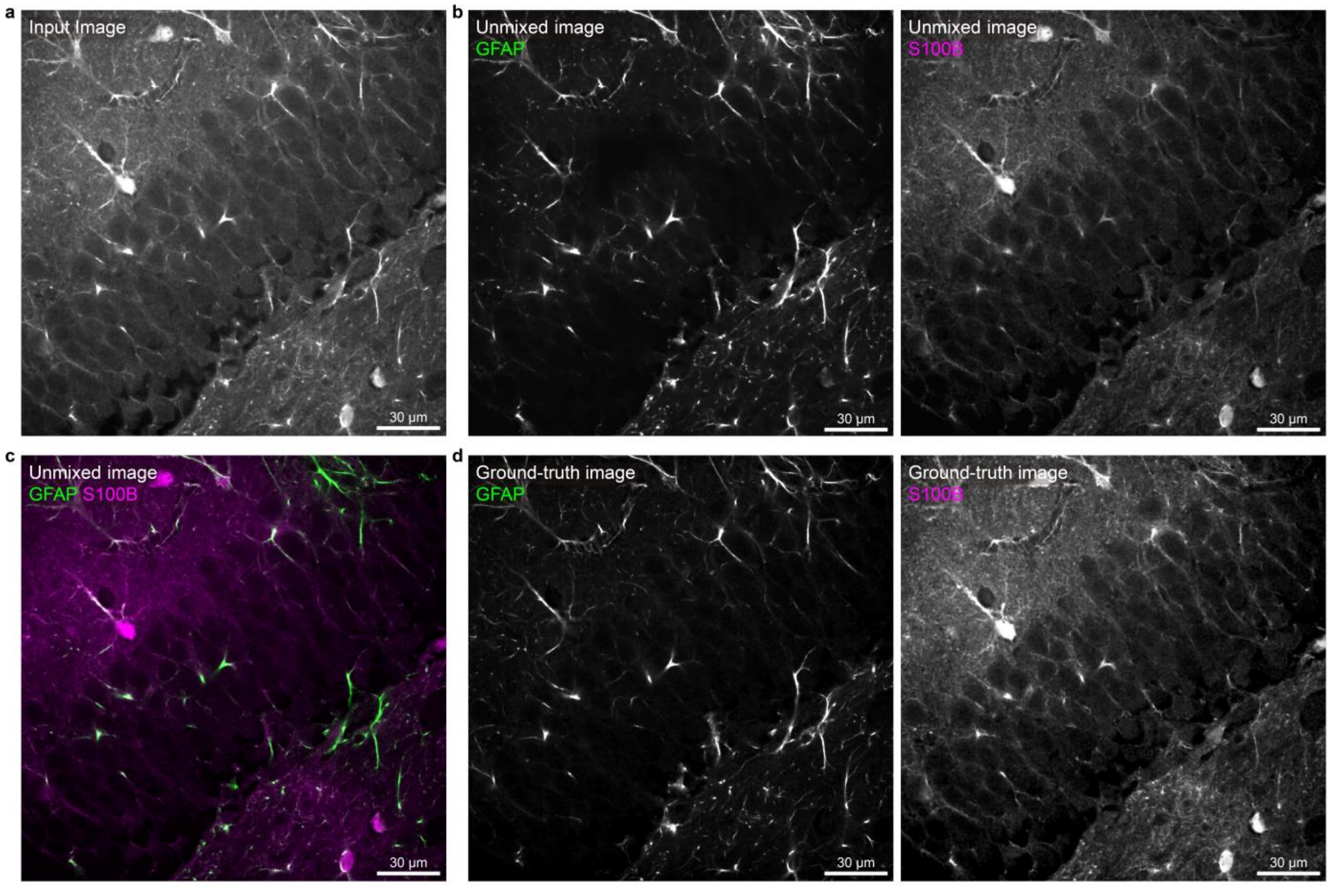

Supplementary Figure 11. Visualization of the unmixing results: GFAP and S100B. a, Input gray scale image. **b**, Unmixed individual channel images for each protein. **c**, Unmixed image represented as channel-wise merge using a green-and-magenta color scheme, with GFAP in green and S100B in magenta. **d**, Ground-truth image of each protein. Scale bar = 30 µm in **a-d**.

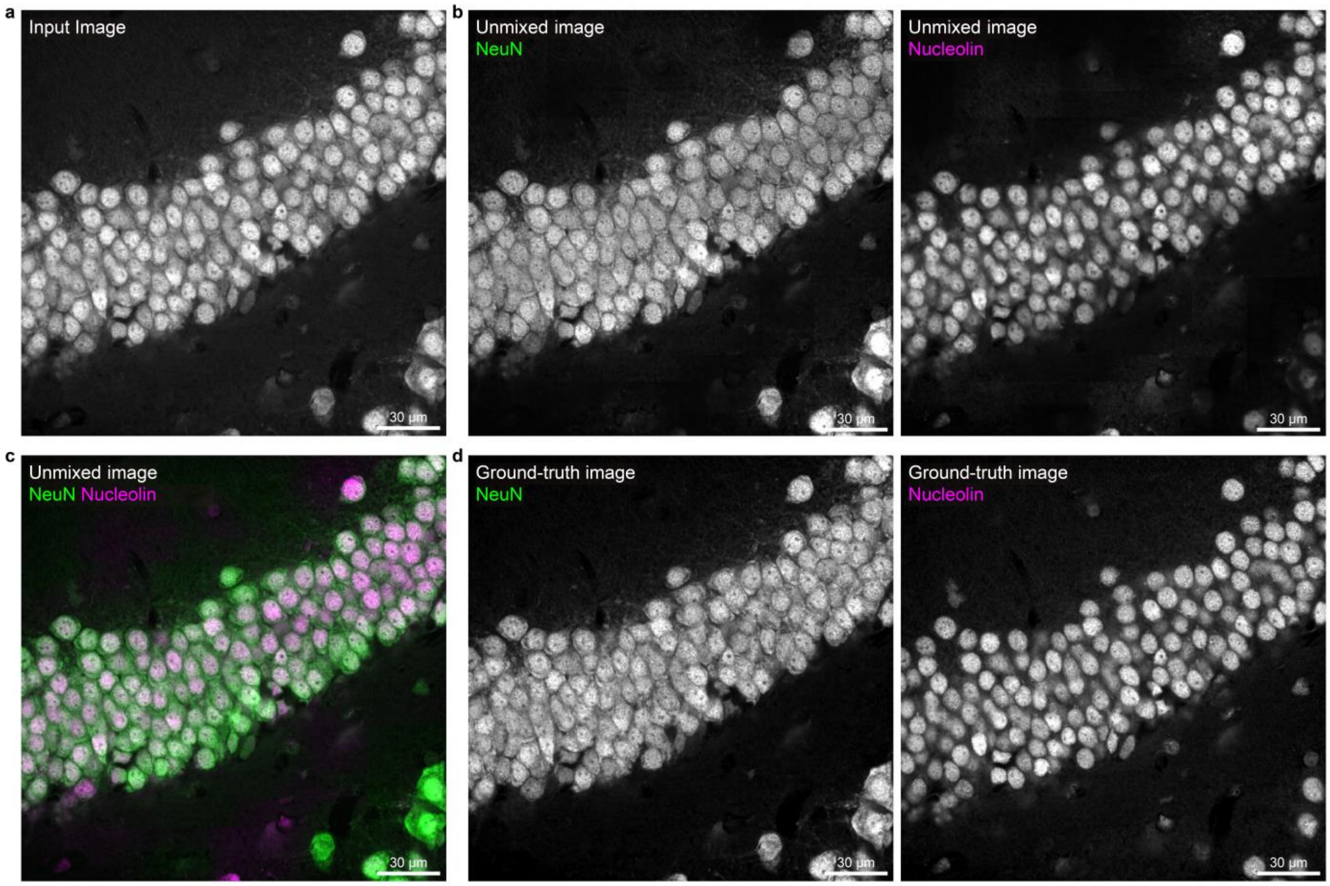

Supplementary Figure 12. Visualization of the unmixing results: NeuN and nucleolin. **a**, Input gray scale image. **b**, Unmixed individual channel images for each protein. **c**, Unmixed image represented as channel-wise merge using a green-and-magenta color scheme, with NeuN in green and nucleolin in magenta. **d**, Ground-truth image of each protein. Scale bar = 30 µm in **a-d**.

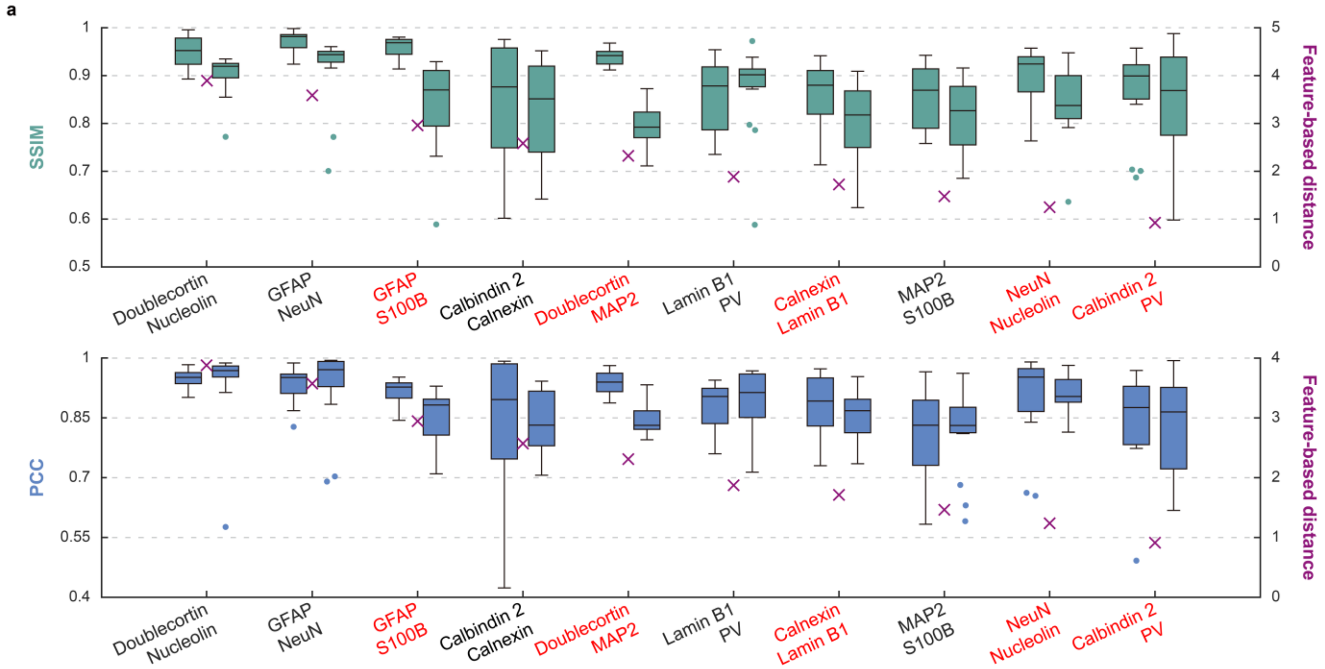

Supplementary Figure 13. Unmixing performance of individual proteins. a, Comparison of between feature-based distance and unmixing performance using SSIM and PCC as metrics. Protein pairs belonging to optimal grouping, group 1, are labeled in black on the axis, while protein pairs belonging to alternative grouping, group 2, are labeled in red. For each protein pair, unmixing performance of individual proteins were displayed as box-and-whisker plots for non-overlapping patches ($n = 16$), with the feature-based distances shown as a purple x marker. The boxes show the interquartile range (IQR) with the median, while whiskers extend to 1.5 times the IQR. Individual points represent outliers, defined as values falling outside the whiskers.

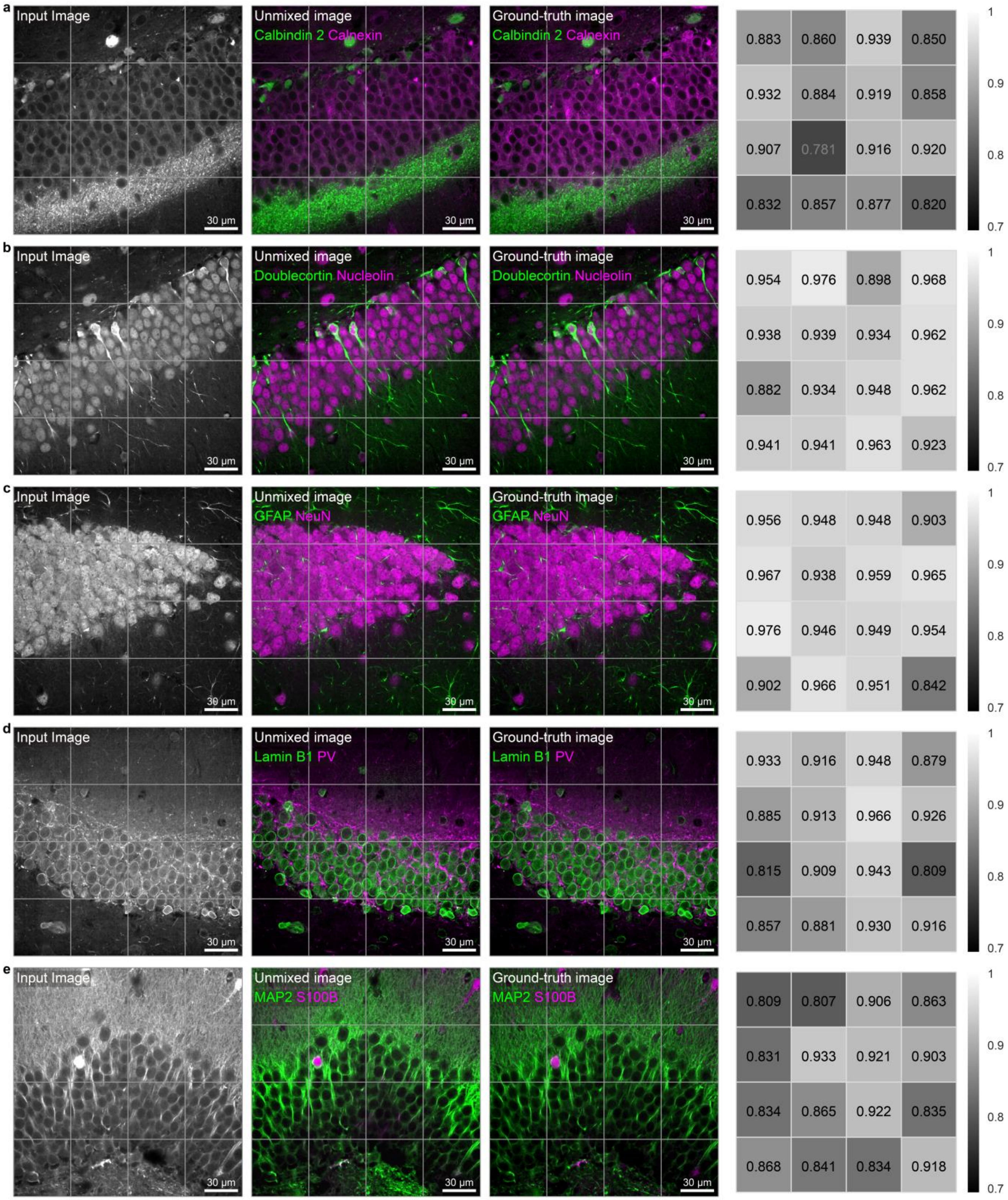

Supplementary Figure 14. Spatial analysis of unmixing results: group 1. a-e, For each protein pair in group 1, we selected representative z-positions from the complete z-stack and divided the field of view into a 4×4 grid to evaluate local unmixing performance. For each grid cell, we calculated the Structural Similarity Index Measure (SSIM) between unmixed result and

ground truth image. From left to right: grayscale input image, unmixed result, ground truth image, and grid-wise SSIM map. Images were displayed with representative z position while the grid-wise SSIM map was calculated for represented z position. Both unmixed and ground truth images are shown as two-channel merges using a green-magenta color scheme. **a**, Results of (calbindin 2, calnexin) pair, with calbindin 2 in green and calnexin in magenta; **b**, Results of (doublecortin, nucleolin) pair, with doublecortin in green and nucleolin in magenta; **c**, The results of (GFAP, NeuN) pair, with GFAP in green and NeuN in magenta; **d**, The results of (lamin B1, PV) pair, with lamin B1in green and PV in magenta; **e**, The results of (MAP2, S100B) pair, with MAP2 in green and S100B in magenta. Scale bar = 30 μm in **a-e**.

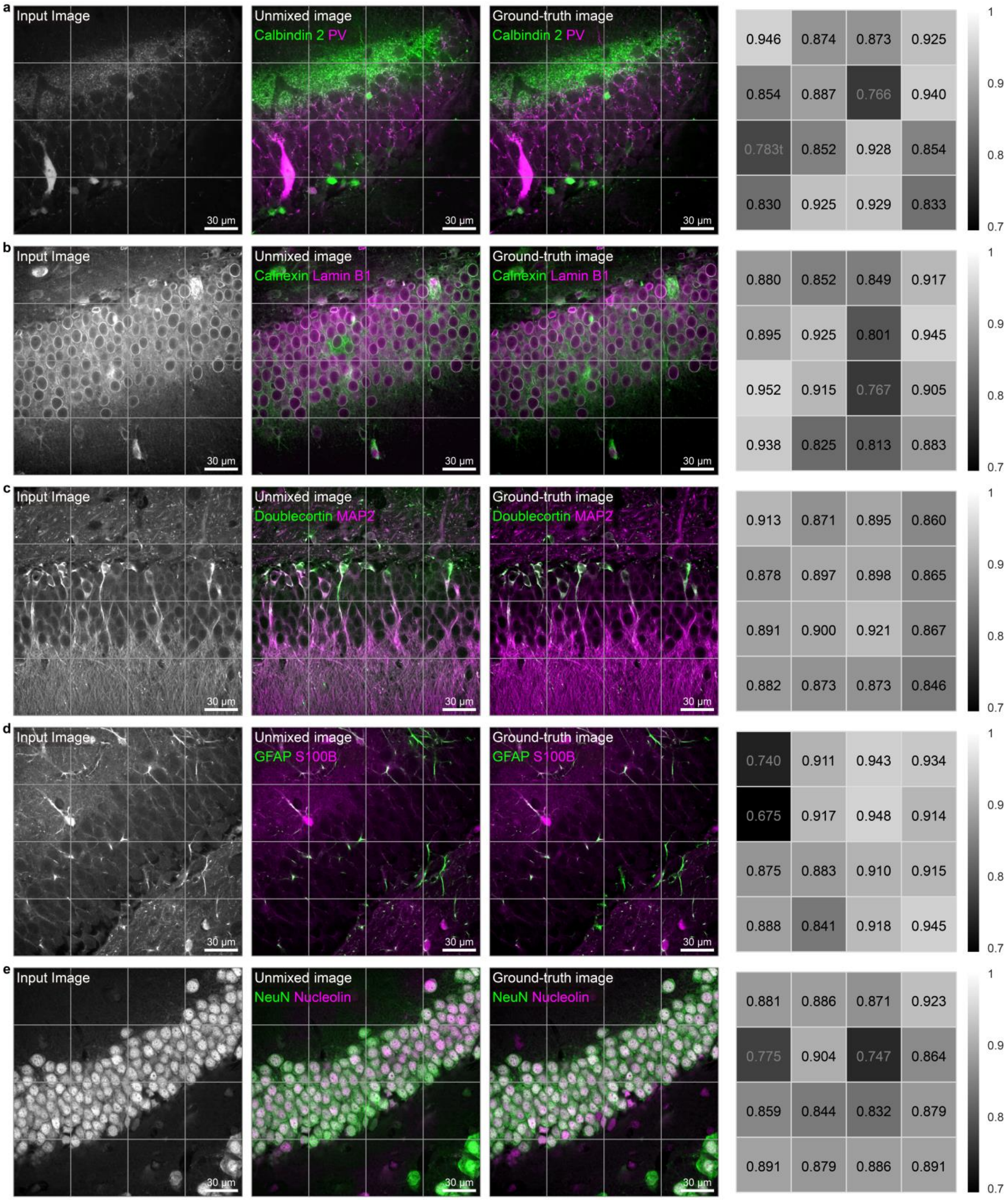

Supplementary Figure 15. Spatial analysis of unmixing results: group 2. a-e, For each protein pair in group 2, we selected representative z-positions from the complete z-stack and divided the field of view into a 4×4 grid to evaluate local unmixing performance. From left to right: grayscale input image, unmixed result, ground truth image, and grid-wise SSIM map. Images

were displayed with representative z position while the grid-wise SSIM map was calculated for represented z position. Both unmixed and ground truth images are shown as two-channel merges using a green-magenta color scheme. **a**, Results of (calbindin 2, PV) pair, with calbindin 2 in green and PV in magenta; **b**, Results of (calnexin, lamin B1) pair, with calnexin in green and lamin B1 in magenta; **c**, Results of (doublecortin, MAP2) pair, with doublecortin in green and MAP2 in magenta; **d**, Results of (GFAP, S100B) pair, with GFAP in green and S100B in magenta; **e**, Results of (NeuN, nucleolin) pair, with NeuN in green and nucleolin in magenta. Scale bar = 30 μm in **a-e**.

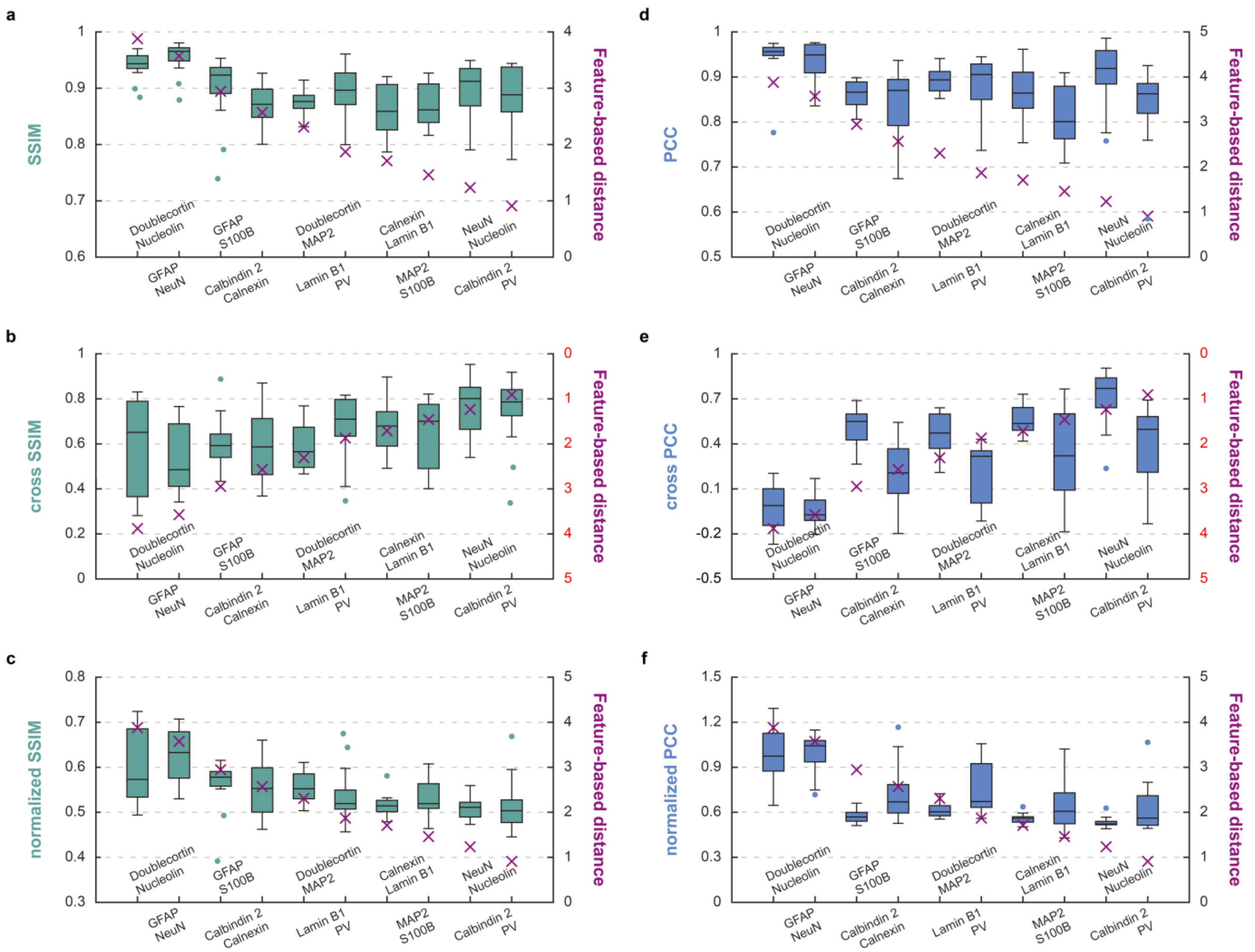

Supplementary Figure 16. Relationship between feature-based distances and unmixing performance metrics. a-f, Correlation analysis between feature-based distance and unmixing performance using SSIM and PCC as metrics. Each box-and-whisker plot represents the SSIM or PCC values of non-overlapping patches ($n = 16$) for the corresponding protein pair, with the feature-based distances shown as a purple x marker. The boxes show the interquartile range (IQR) with the median, while whiskers extend to 1.5 times the IQR. Individual points represent outliers, defined as values falling outside the whiskers. **a**, Correlation analysis between feature-based distances and SSIM values for unmixed protein pairs, demonstrating the predictive power of feature-based distances for unmixing performance with the Pearson correlation coefficient of 0.7144 for average SSIM and 0.6873 for median SSIM; **b**, Cross-similarity analysis showing the negative correlation between cross-SSIM and feature-based distances with the Pearson correlation coefficient of -0.8504 for average cross SSIM and -0.7788 for median cross SSIM, where the feature-based distance axis in inverted and displayed in red; **c**, Normalized SSIM analysis (normalized by $\frac{\text{SSIM}}{1 + \text{cross SSIM}}$ to avoid large deviations from near-zero denominators) revealing enhanced correlation with feature-based distances, yielding a Pearson correlation coefficient of 0.9404 with average normalized SSIM and 0.8942 with median normalized SSIM, validating the effectiveness of our feature-based approach in predicting unmixing performance; **d**, PCC analysis exhibiting comparable trends to SSIM, showing positive correlation with feature-based distances with the Pearson correlation coefficient of 0.6212 for average PCC and 0.6024 for median PCC; **e**, Cross-PCC analysis exhibiting negative correlation between feature-based distances, showing a Pearson correlation coefficient of -0.6990 with average cross PCC and -0.7411 with median cross PCC, where the feature-based distance axis in inverted and displayed in red; **f**, Normalized PCC analysis (normalized by $\frac{\text{PCC}}{1 + \text{cross PCC}}$ to avoid large deviations from near-zero denominators) showing improved correlation

with feature-based distances, obtaining the Pearson correlation coefficient of 0.7699 for average normalized PCC and 0.8145 for median normalized PCC.

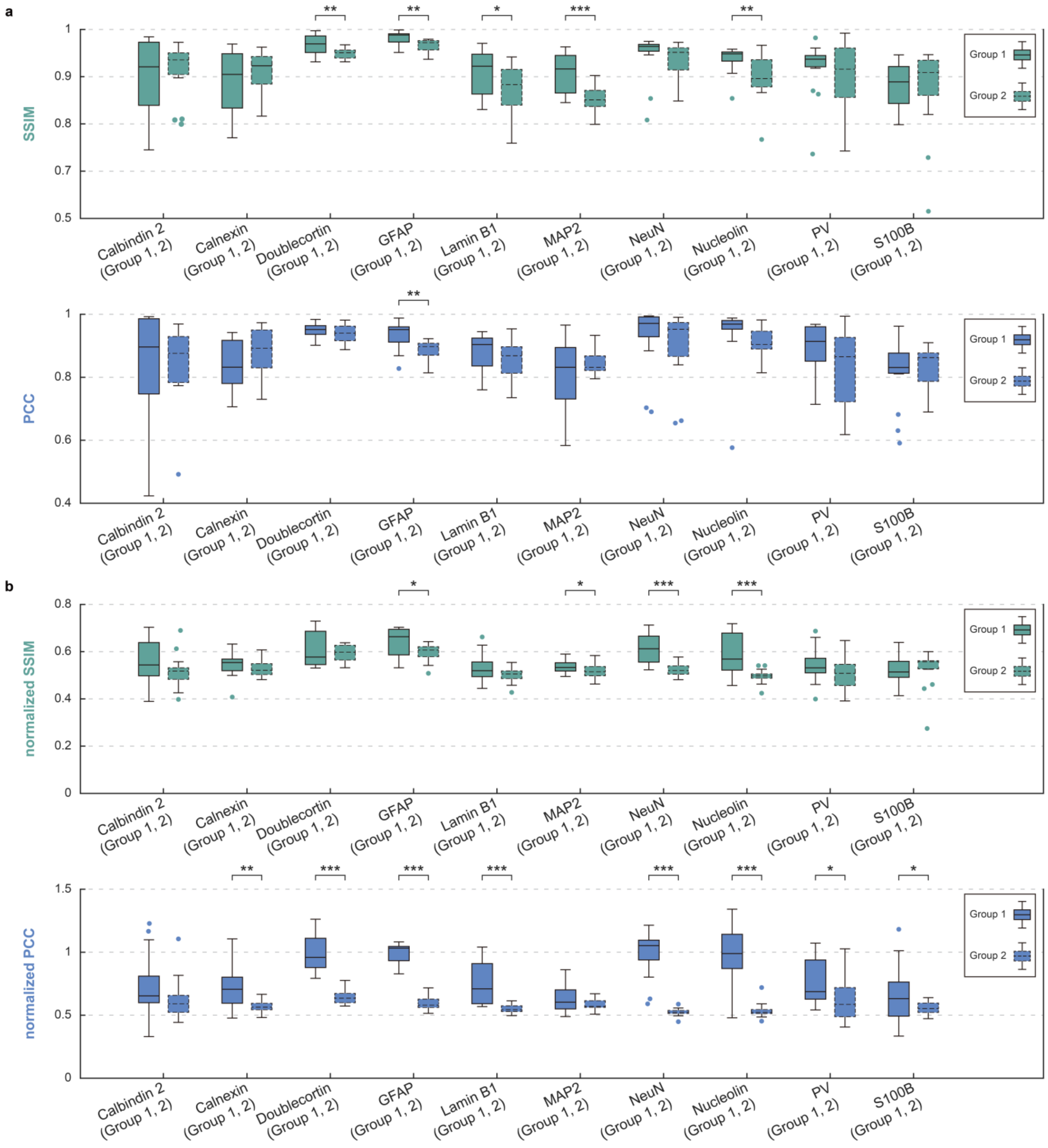

Supplementary Figure 17. Statistical comparison of unmixing performance between optimal grouping (group 1) and alternative grouping (group 2). a, Comparison of unmixing performance using SSIM (top) and PCC (bottom) for each protein in different groupings. Box-and-whisker plots show the unmixing performance values across non-overlapping patches ($n = 16$). A two-sided paired-sample t-test was used. **b**, Comparison of unmixing performance using normalized SSIM (top) and normalized PCC (bottom), which adjusts raw performance by the cross-similarity between paired proteins, for each protein in different grouping. Box-and-whisker plots show the unmixing performance values across non-overlapping patches ($n = 16$).

A two-sided paired-sample t-test was used. (*$p < 0.1$, **$p < 0.01$, ***$p < 0.001$).

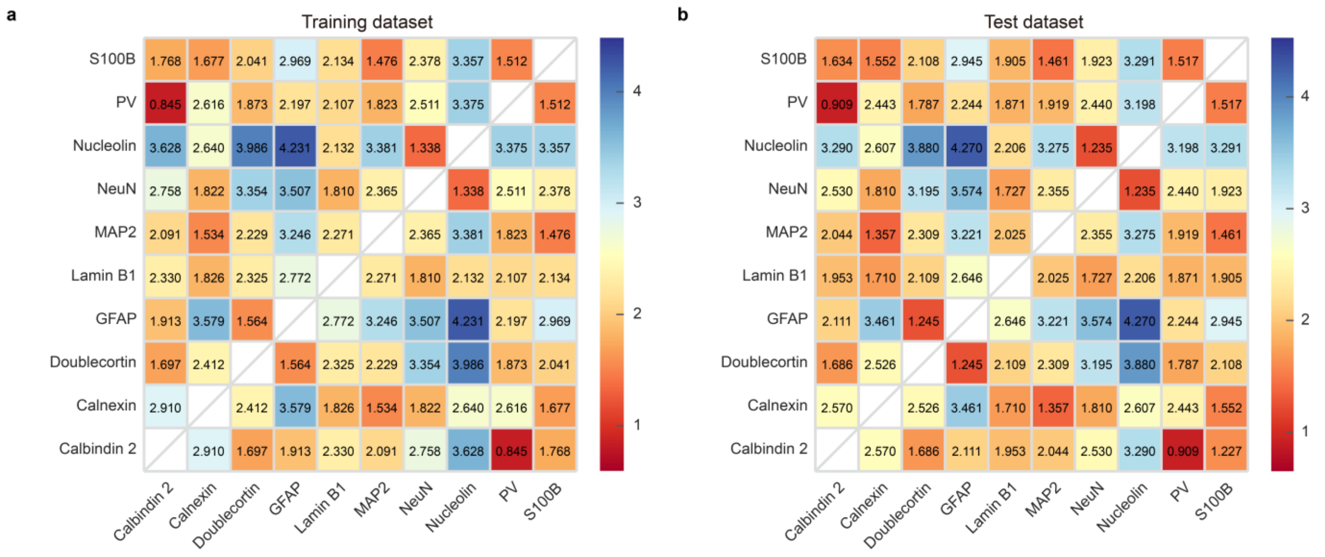

Supplementary Figure 18. Comparison of feature-based distance matrices between the separate training and test datasets. a-b, The feature-based distance matrices calculated from two separate datasets are displayed as heatmaps with a red-yellow-blue color scheme, where blue indicates higher and red indicates lower feature-based distances. For each protein, three z-stacks were used for training data and one z-stack was used for test data, ensuring complete separation between them. **a**, Training dataset; **b**, Test dataset. The matrices show pairwise feature-based distances between various protein markers: calbindin2, calnexin, doublecortin, GFAP, lamin B1, MAP2, NeuN, nucleolin, PV, and S100B. The strong agreement between training and test matrices with a Pearson correlation coefficient of 0.9923 demonstrates that feature extraction remains stable across different samples.

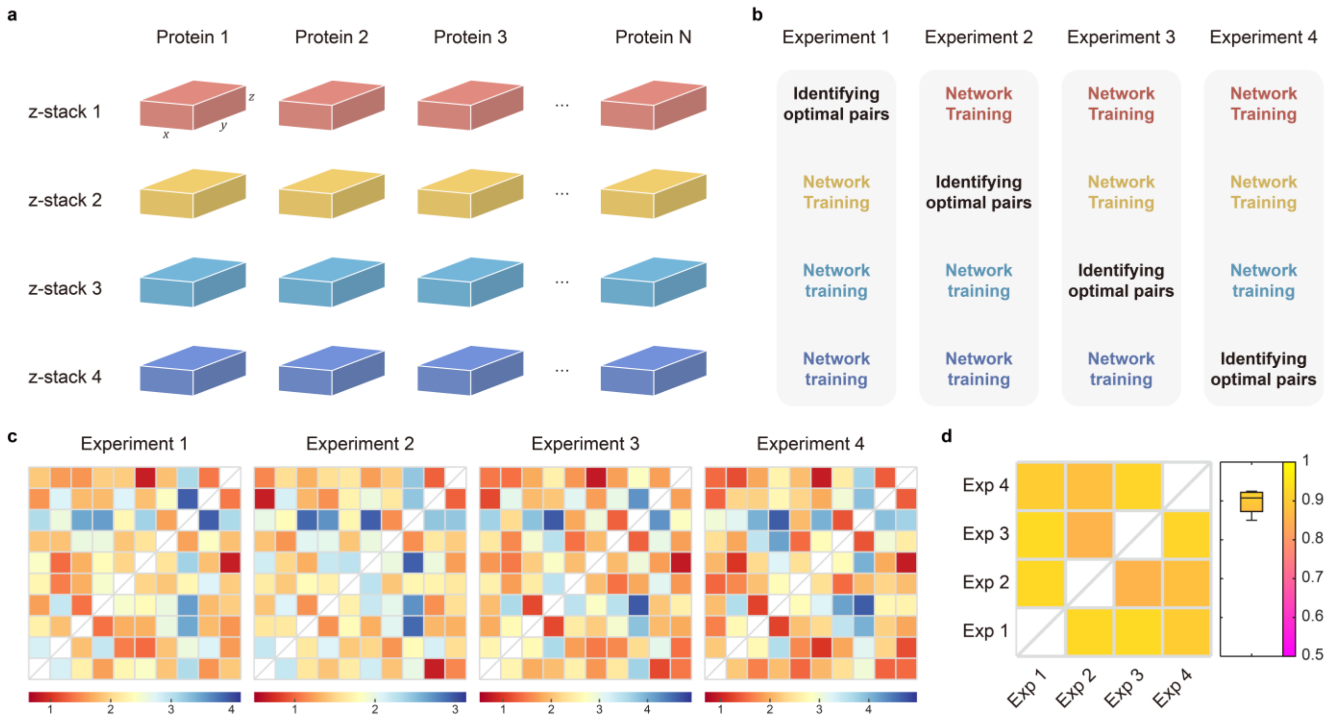

Supplementary Figure 19. Cross-validation on the reproducibility of feature extraction across different dataset combinations. a, Schematic representation of the z-stack datasets. Each protein (1 to N) has four z-stack images, where each z-stack consists of multiple image slices. Each z-stack of protein images is shown in different colors to indicate separate imaging sessions. For each protein, three z-stacks were used to train the feature extraction network, while one was used to identify optimal pairs. **b**, Overview of the cross-validation scheme, showing how four z-stack images were alternately used for network training (colored text) and optimal pair identification (black text) across different experiments. **c**, The pairwise feature-based distance matrices calculated from each experiment, are displayed as heatmaps with a red-yellow-blue color scheme, where blue indicates higher and red indicates lower feature-based distances between protein pairs. The consistency in relative patterns across matrices demonstrates the robustness of our approach across different experiments. **d**, Cross-correlation analysis between feature-based distance matrices, with the heatmap showing the correlation between experiments (left) and their distribution summarized in a box-and-whisker plot (right, $n = 6$). Yellow indicates a higher correlation, while pink indicates a lower correlation. The boxes show the interquartile range (IQR) with the median, while whiskers extend to 1.5 times the IQR. Individual points represent outliers, defined as values falling outside the whiskers. The correlation values above 0.8 confirm that the relative relationships between protein pairs are preserved regardless of the training and optimal pair identification dataset combinations.

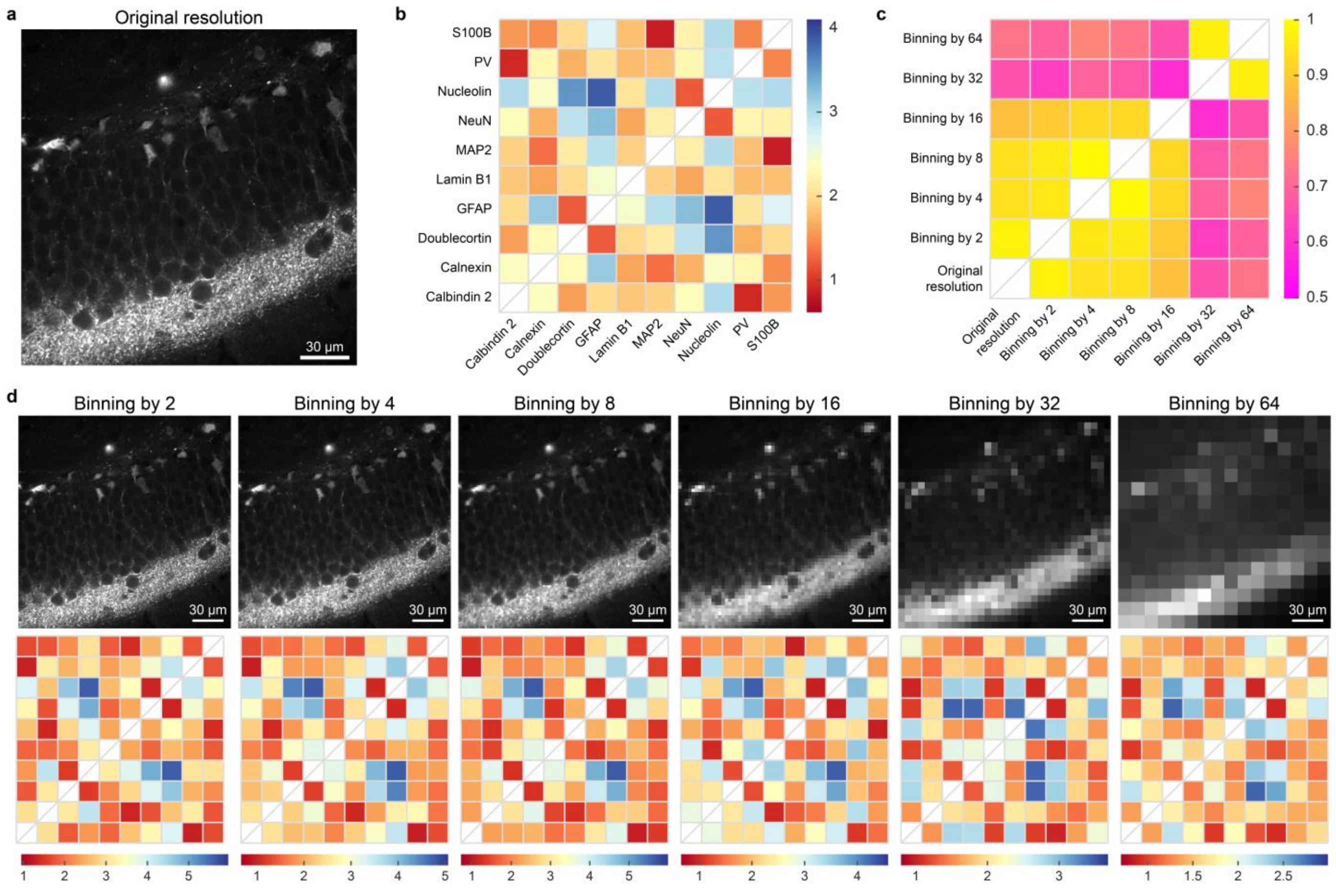

Supplementary Figure 20. Effects of spatial resolution on feature-based distance matrices. a, Original resolution image showing the spatial expression pattern of calbindin 2 as a representative example among the 10 protein markers. **b**, The pairwise feature-based distance matrix calculated from the feature extraction network for original resolution images of 10 protein markers (calbindin 2, calnexin, doublecortin, GFAP, lamin B1, MAP2, NeuN, nucleolin, PV, S100B), displayed as a heatmap with a red-yellow-blue color scheme, where blue indicates higher and red indicates lower feature-based distances between protein pairs. **c**, Cross-correlation analysis between feature-based distance matrices at different binning factors (2, 4, 8, 16, 32, and 64), showing the relationship between different spatial resolutions. Yellow indicates a higher correlation, while pink indicates a lower correlation. **d**, Representative images of calbindin 2 showing the spatial resolution with different binning factors. The corresponding pairwise feature-based distance matrices from individually trained feature extraction networks with each condition are shown below. Scale bar = 30 μm in **a** and **d**.

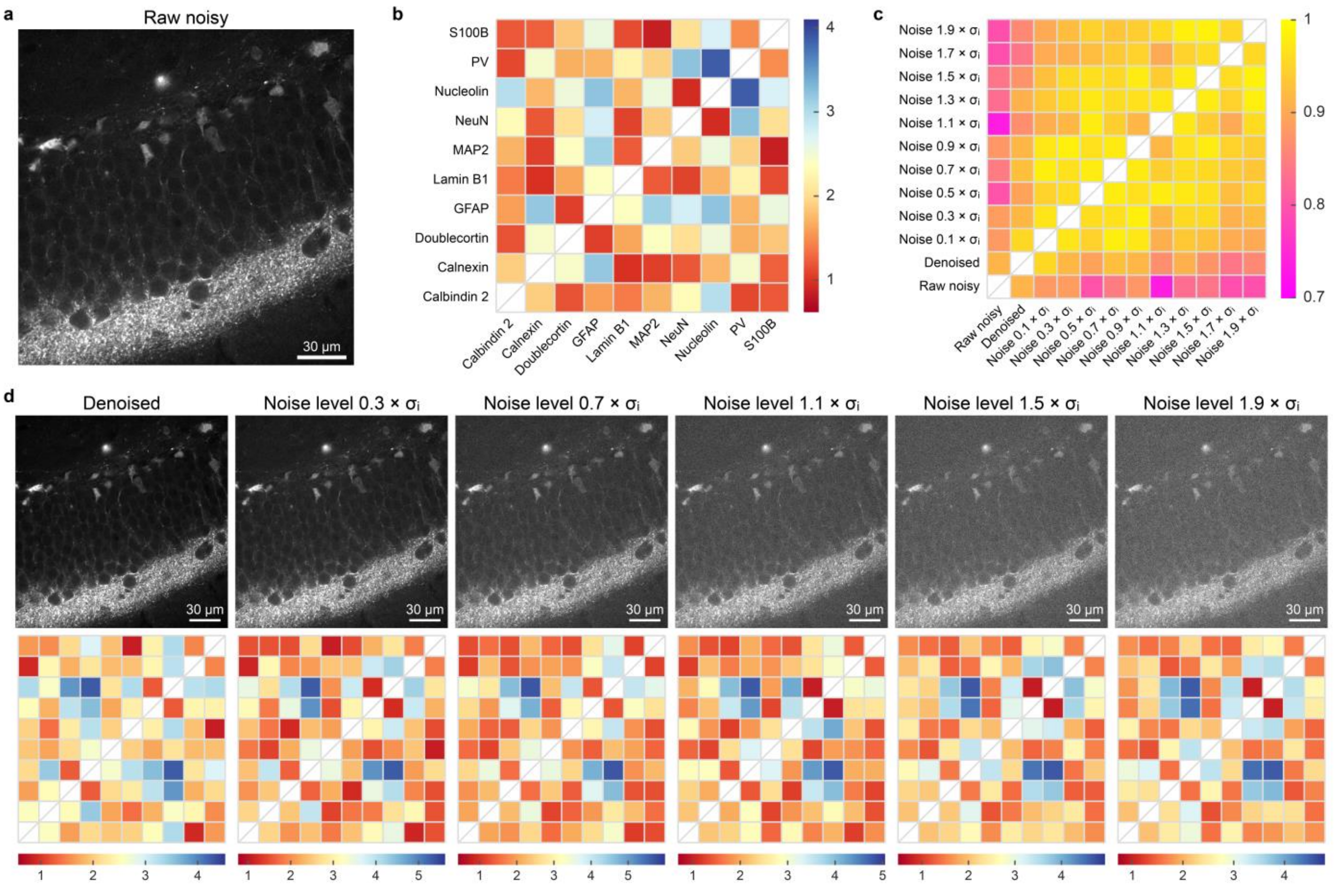

Supplementary Figure 21. Effects of noise levels on feature-based distance matrices. a, Raw noisy image showing the spatial expression pattern of calbindin 2 as a representative example among the 10 protein markers. **b**, The pairwise feature-based distance matrix calculated from the feature extraction network for raw noisy images of 10 protein markers (calbindin 2, calnexin, doublecortin, GFAP, lamin B1, MAP2, NeuN, nucleolin, PV, S100B), displayed as a heatmap with a red-yellow-blue color scheme, where blue indicates higher and red indicates lower feature-based distances between protein pairs. **c**, Cross-correlation analysis between feature-based distance matrices at different noise levels, showing the relationship between raw noisy, denoised, and noise-added conditions. Yellow indicates a higher correlation, while pink indicates a lower correlation. **d**, Representative images of Calbindin 2 showing the noise level of denoised and incremental Gaussian noise added images ($k \times \sigma_i$, $k =$ 0.3 to 1.9, where σ_i represents the standard deviation of the denoised image). The corresponding pairwise feature-based distance matrices from individually trained feature extraction networks with each condition are shown below. Scale bar = 30 µm in **a** and **d**.

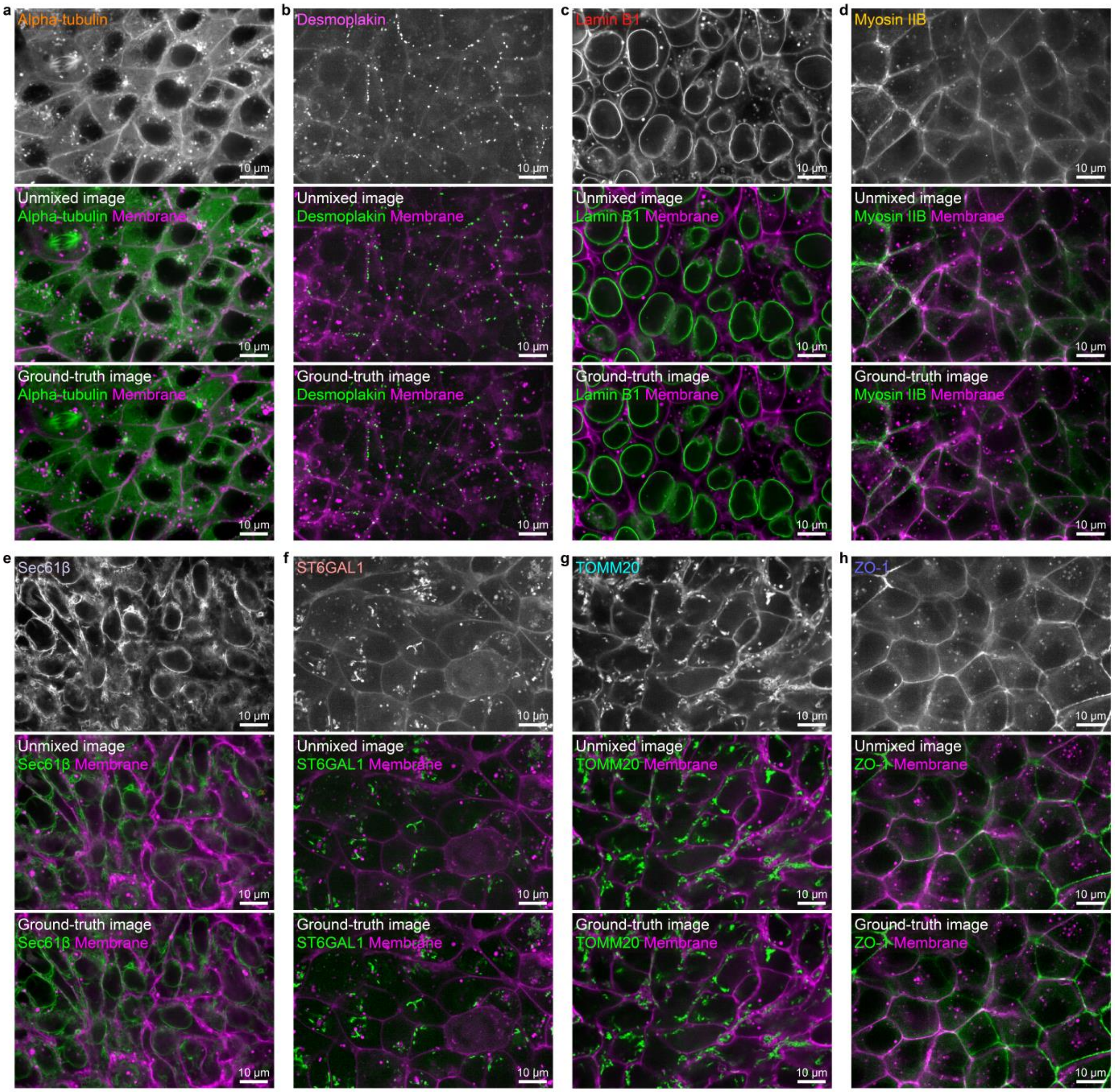

Supplementary Figure 22. Unmixing results of the public human cell dataset from Allen Institute for Cell Science: proteins paired with cell membrane. a-h, Representative image showing gray scale network input (synthetic images) and corresponding channel-wise merged network output (unmixed images) using a green-and-magenta color scheme. Coefficients of 0.5 were utilized for synthetic image generation, which represents an equal contribution from each protein image in synthetic mixed images. For each pair, the target protein is shown in green and the cell membrane in magenta. Protein pairs shown: **a**, (alpha-tubulin, cell membrane); **b**, (desmoplakin, cell membrane); **c**, (lamin B1, cell membrane); **d**, (myosin IIB, cell membrane); **e**, (Sec61β, cell membrane); **f**, (ST6GAL1, cell membrane); **g**, (TOMM20, cell membrane); **h**, (ZO-1, cell membrane). Scale bar = 10 μm in **a-h**.

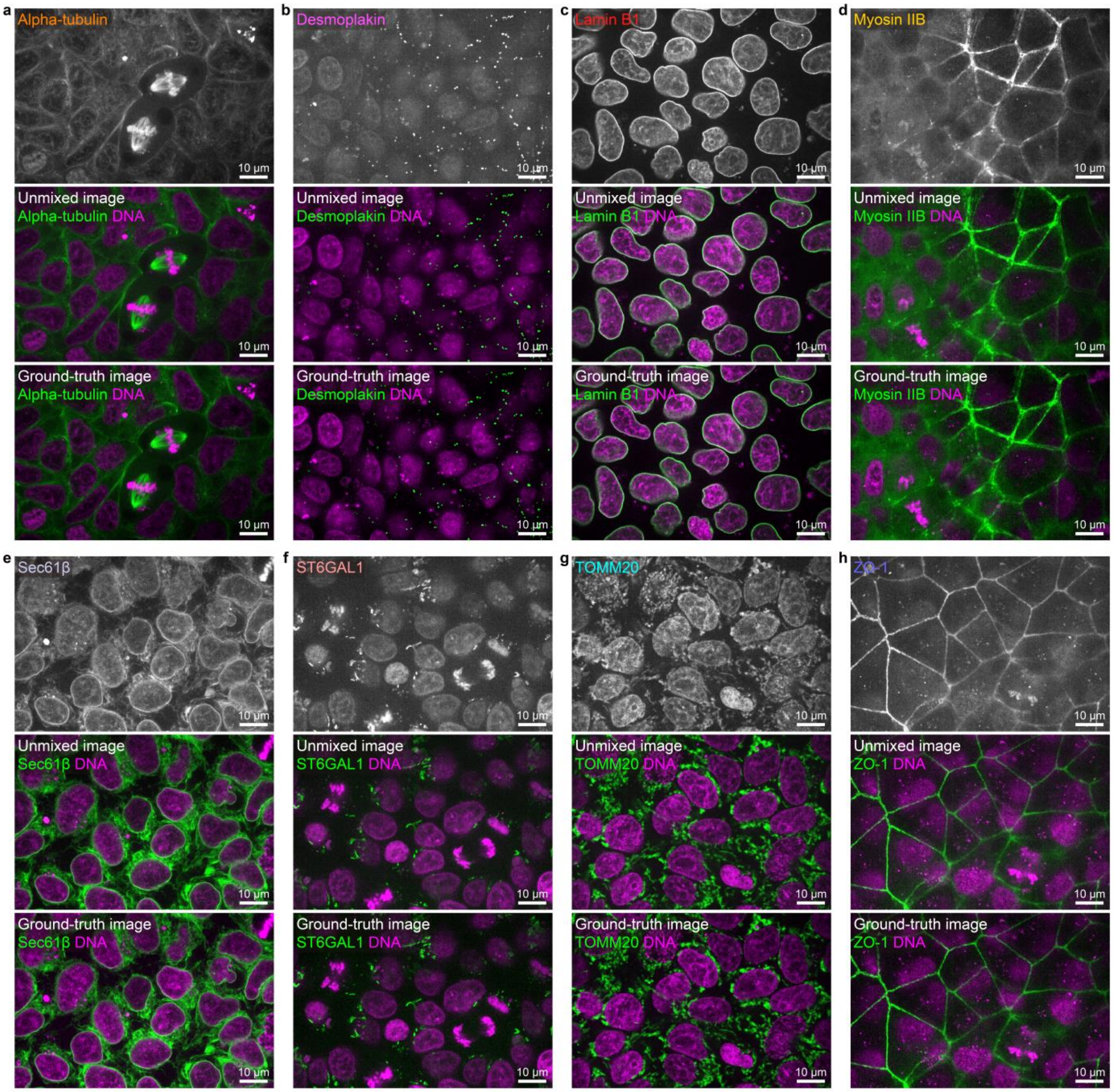

Supplementary Figure 23. Unmixing results of the public human cell dataset from Allen Institute for Cell Science: proteins paired with DNA. a-h, Representative image showing gray scale network input (synthetic images) and corresponding channel-wise merged network output (unmixed images) using a green-and-magenta color scheme. Coefficients of 0.5 were utilized for synthetic image generation, which represents an equal contribution from each protein image in synthetic mixed images. For each pair, the target protein is shown in green and the DNA in magenta. Protein pairs shown: **a**, (alpha-tubulin, DNA); **b**, (desmoplakin, DNA); **c**, (lamin B1, DNA); **d**, (myosin IIB, DNA); **e**, (Sec61β, DNA); **f**, (ST6GAL1, DNA); **g**, (TOMM20, DNA); **h**, (ZO-1, DNA). Scale bar = 10 µm in **a-h**.

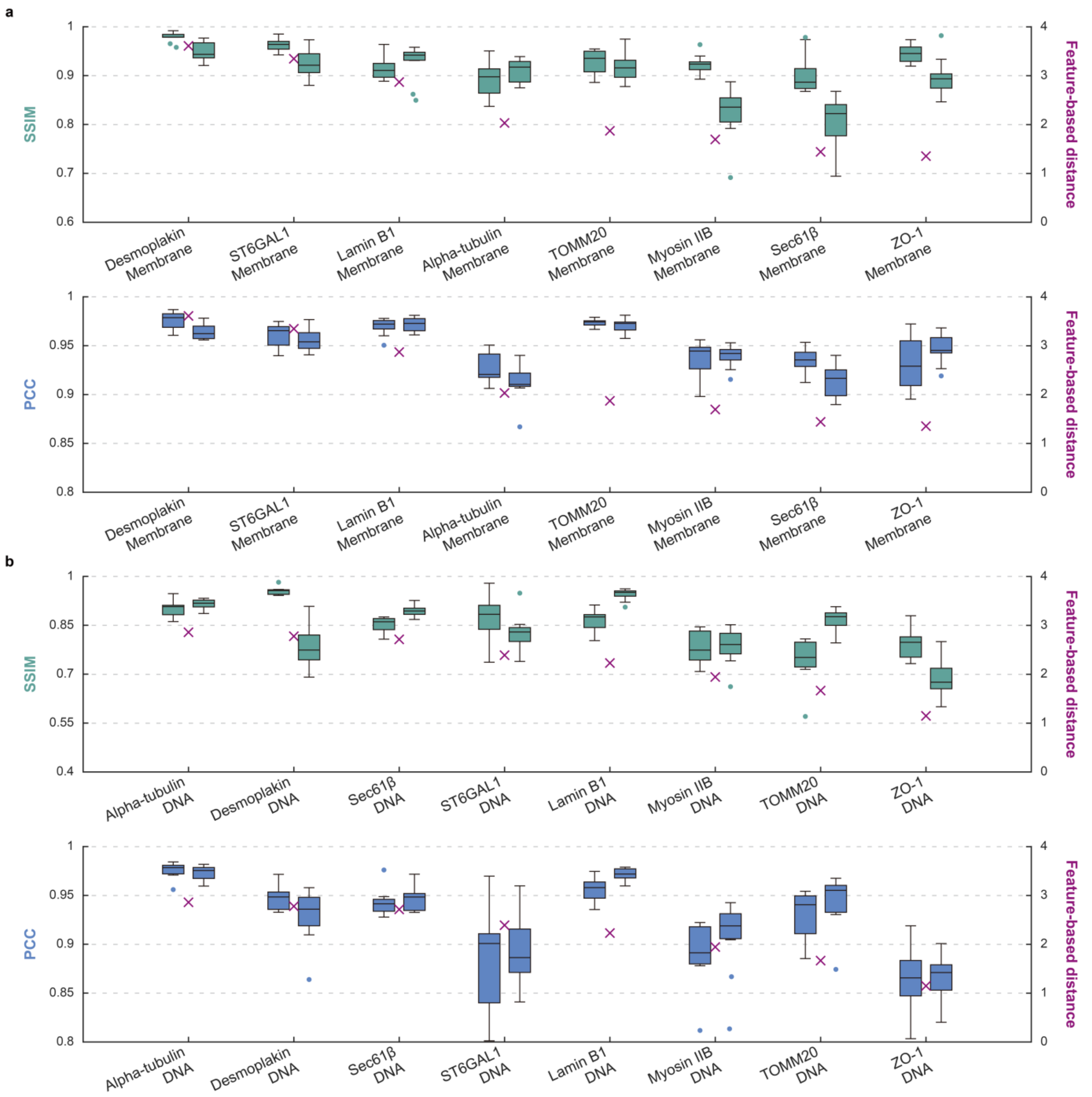

Supplementary Figure 24. Unmixing performance of individual proteins for the public human cell dataset from Allen Institute for Cell Science. a-b, Comparison of between feature-based distance and unmixing performance using SSIM and PCC as metrics. For each protein pair, unmixing performance of individual proteins were displayed as box-and-whisker plots for non-overlapping patches ($n = 16$), with the feature-based distances shown as a purple x marker. The boxes show the interquartile range (IQR) with the median, while whiskers extend to 1.5 times the IQR. Individual points represent outliers, defined as values falling outside the whiskers. **a**, Unmixing performance of 8 proteins paired with membrane, where the left box in each pair shows the unmixing performance of the protein paired with membrane and the right box shows the unmixing performance of the membrane; **b**, Unmixing performance of 8 proteins paired with DNA, where the left box in each pair shows the unmixing performance of the protein paired with DNA and the right box shows the unmixing performance of the DNA.

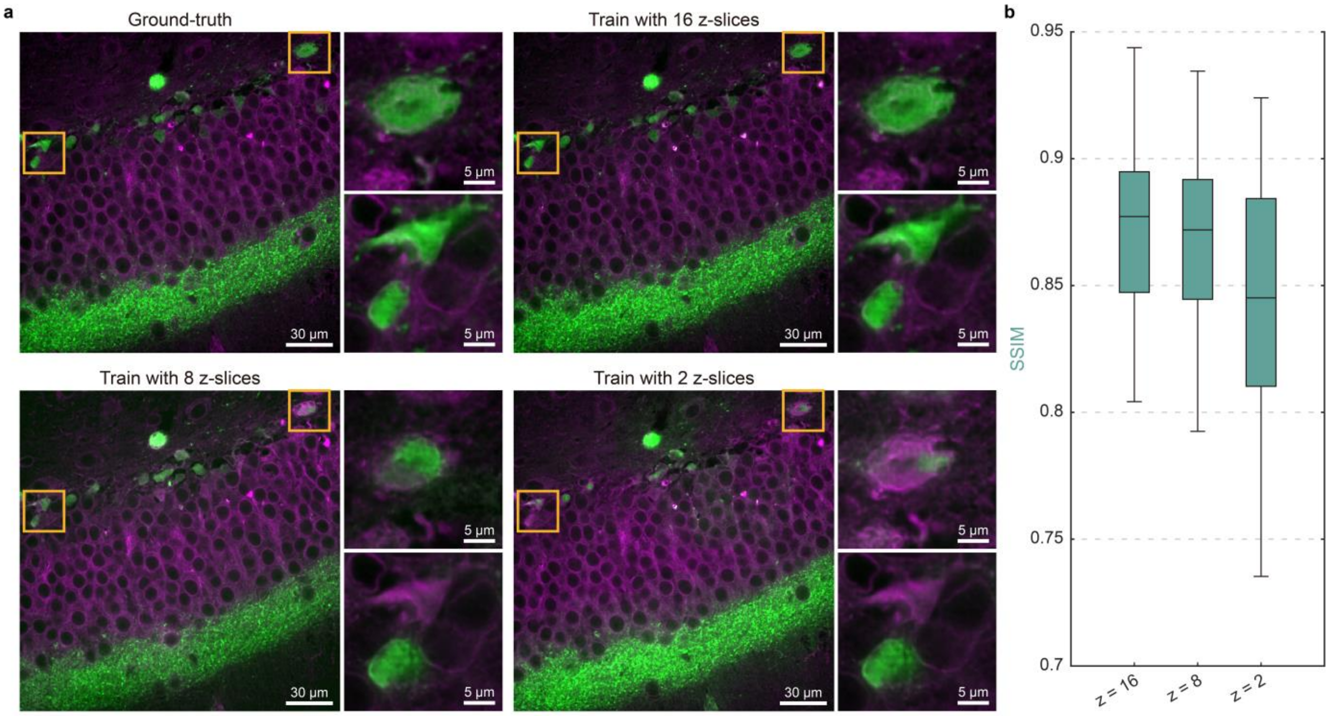

Supplementary Figure 25. Comparison of unmixing performance of 3D-trained models across varying z-stack depths. Analysis of z-stack depth effects on protein unmixing performance, demonstrated with (calbindin 2, calnexin) pair as representative protein pair. **a**, Ground truth and unmixing results at a single z-position shown in channel-wise merge using a green-and-magenta color scheme (calbindin 2 in green, calnexin in magenta) for 3D-trained models across varying z-stack depths (16, 8, and 2 z-slices). Magnified views of regions highlighted by yellow boxes are shown on the right of each condition. **b**, Box-and-whisker plots of unmixing performance quantified by SSIM demonstrating decreased performance with reduced z-depth ($n = 16$, non-overlapping 3-dimensional z-stack patches). The boxes show the interquartile range (IQR) with the median, while whiskers extend to 1.5 times the IQR. Scale bar = 30 μm in full-field images and 5 μm in magnified views in **a**.

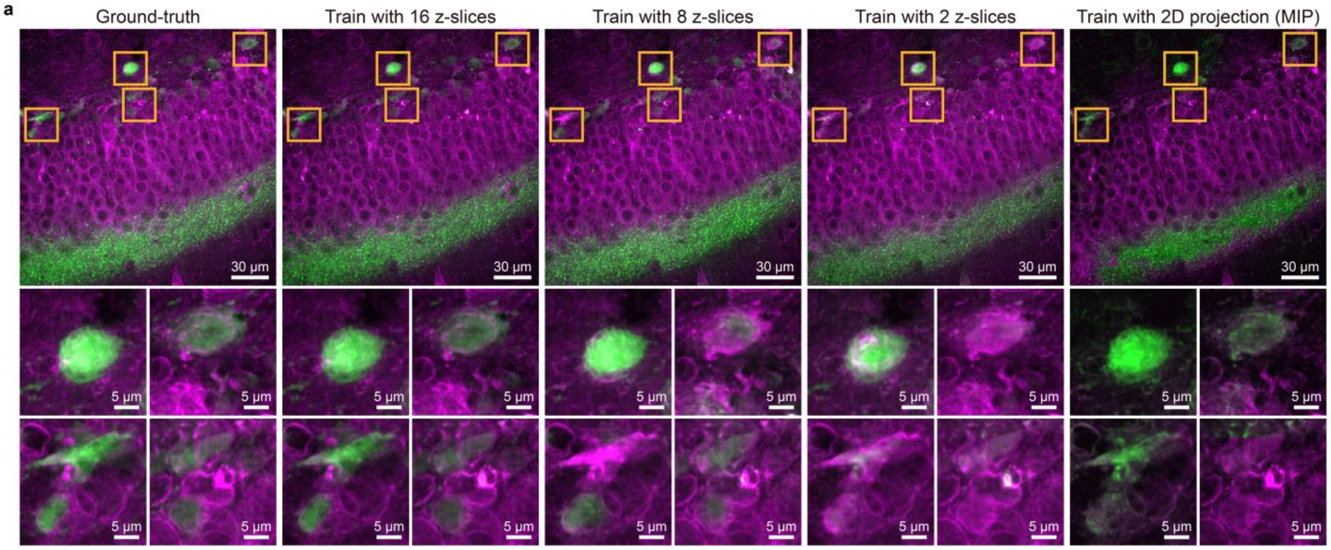

Supplementary Figure 26. Comparison of 3D-trained models with 2D projection trained model. Demonstration of 3D-trained model advantages using (calbindin 2, calnexin) pair as a representative protein pair. **a**, Maximum intensity projections (MIPs) of ground truth and unmixing results shown in channel-wise merge using a green-and-magenta color scheme (calbindin 2 in green, calnexin in magenta) comparing 3D-trained models with varying z-stack depths (16, 8, and 2 z-slices) to 2D projection trained model. Magnified views of regions highlighted by yellow boxes are shown below each condition. Scale bar = 30 μm in full-field images and 5 μm in magnified views in **a**.

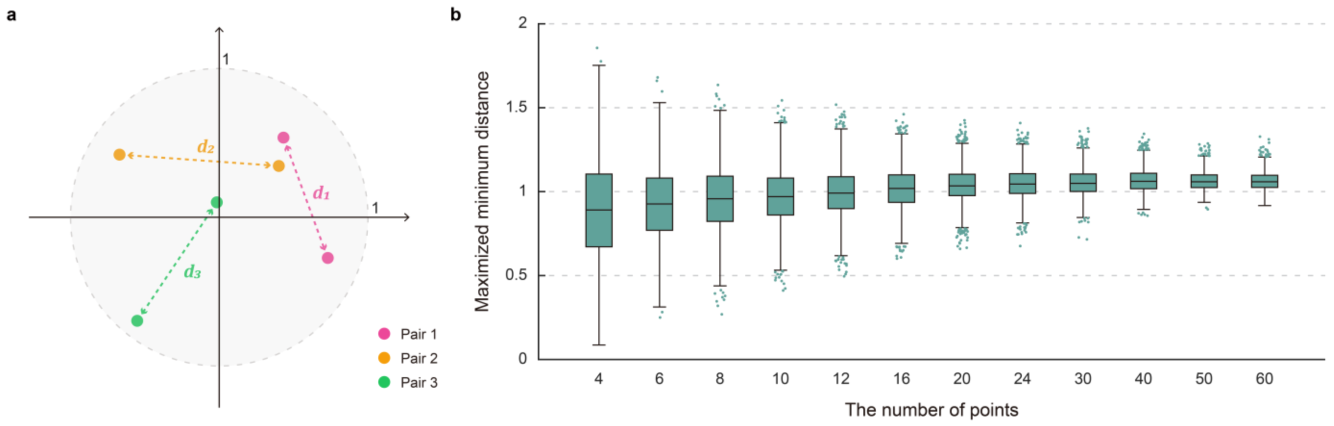

Supplementary Figure 27. Scalability analysis of SEPARATE with an increasing number of protein markers through Monte Carlo experiments. a, Illustration of the Monte Carlo experiment setup. Random points ($k = 6$) are generated within a unit circle, and optimal grouping that maximizes the minimum distance between paired points is identified. The paired points are connected by dashed lines, with distances d_1, d_2, and d_3. **b**, Box-and-whisker plots of maximized minimum distance for 2,000 independent trials with an increased number of points ($k = 4$ to 60). The boxes show the interquartile range (IQR) with the median, while whiskers extend to 1.5 times the IQR. Individual points represent outliers, defined as values falling outside the whiskers. The results show increasing median values and decreasing variance as k increases, demonstrating that SEPARATE achieves more stable and reliable groupings with larger marker sets.

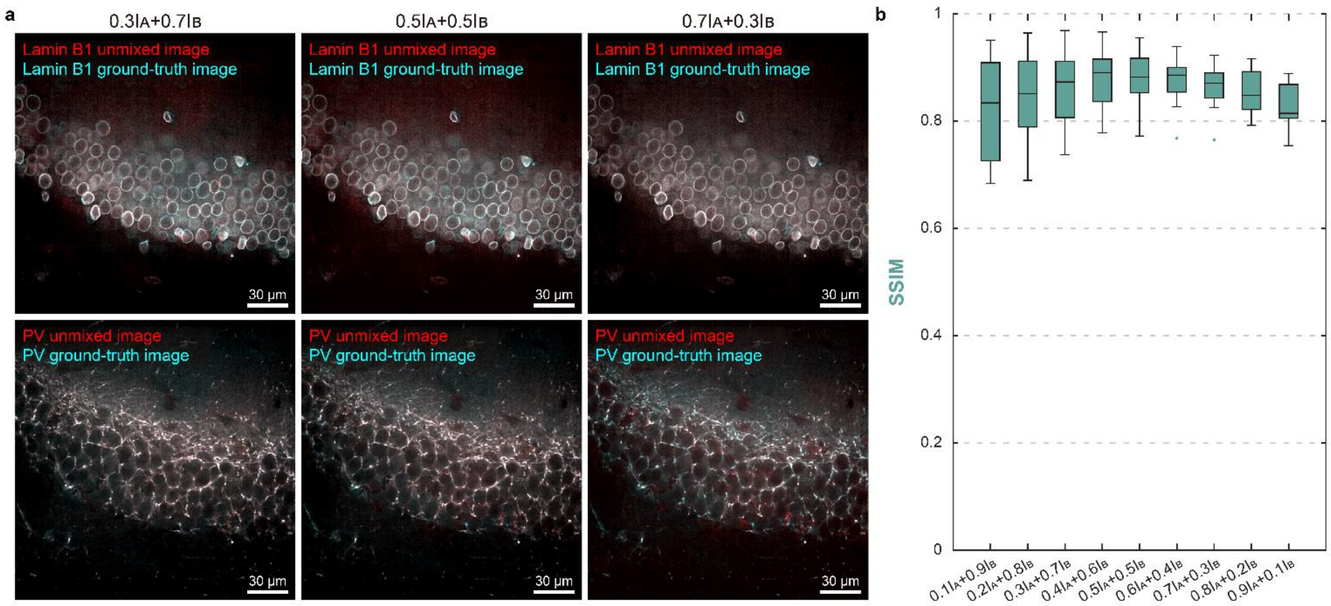

Supplementary Figure 28. Comparison of unmixing performance across different relative brightness of protein signals. **a**, Representative images showing unmixing results for varying signal ratios between two proteins, lamin B1 and PV, for three different mixing coefficients ($0.3\mathrm{I_A} + 0.7\mathrm{I_B}$, $0.5\mathrm{I_A} + 0.5\mathrm{I_B}$, $0.7\mathrm{I_A} + 0.3\mathrm{I_B}$, where $\mathrm{I_A}$ represents the image of lamin B1 and $\mathrm{I_B}$ represents the image of PV). The unmixing results are overlaid with ground-truth images using a red-and-cyan color scheme to enable intuitive interpretation: white indicates correctly unmixed signals, red shows false positive assignments, and cyan represents false negative assignments. Top row shows the comparison between unmixed and ground-truth images for lamin B1, and bottom row shows the comparison for PV. **b**, Quantitative assessment of unmixing performance using SSIM for varying mixing coefficient combination. Each box-and-whisker plot represents the SSIM of non-overlapping patches ($n = 16$) for each signal mixing coefficient. The boxes show the interquartile range (IQR) with the median, while whiskers extend to 1.5 times the IQR. While the performance slightly decreases with larger differences in relative brightness between proteins, the SSIM values consistently remain above 0.7, indicating robust unmixing performance across varying signal ratios. Scale bar = 30 μm in **a**.

SUPPLEMENTARY TABLES

Supplementary Table 1. The reagents used in this study

	Name	Vendor	Catalog #
1	4% paraformaldehyde	T&I	TNI-BPP-9004-500ML
2	10× PBS 7.4 pH	Thermofisher	AM9625
3	Glycine	Sigma-Aldrich	50046
4	sodium azide	Sigma-Aldrich	71289
5	Normal rabbit serum	Jackson immunoresearch	011-000-120
6	Sodium bicarbonate	Sigma-Aldrich	S6297-250G
7	Dimethyl sulfoxide	Sigma-Aldrich	276855-100ml
8	Triton X-100	Sigma	T9284-100ML
9	AffiniPure Fab Fragment Goat Anti-Rabbit IgG, Fc Fragment Specific [1mg]	Jackson immunoresearch	111-007-008
10	NAP DNA Purification Columns	Cytiva	17085302
11	CF®488A Succinimidyl Ester	Biotium	92120
12	CF®568 Succinimidyl Ester	Biotium	92131
13	CF®633 Succinimidyl Ester	Biotium	92133
14	Amicon Ultra-0.5 Centrifugal Filter Unit	Merck Millipore	UFC503096
