# Supplementary material for: Doubling multiplexed imaging capability via spatial expression pattern-guided protein pairing and computational unmixing
Source: Commun Biol. 2025 Jun 14;8:928. doi: 10.1038/s42003-025-08357-5 (PMC12167378; doi:10.1038/s42003-025-08357-5)
Supplement: Supplementary file 2 — Description of Additional Supplementary Materials [file 42003_2025_8357_MOESM2_ESM.pdf]

Description of Additional Supplementary Files

File name: Supplementary Data 1

Description: Numerical source data underlying graphs in main Figures 1-5

File name: Supplementary Data 2

Description: Numerical source data underlying graphs in Supplementary Figures 1-28
