# Supplementary material for: Doubling multiplexed imaging capability via spatial expression pattern-guided protein pairing and computational unmixing
Source: Commun Biol. 2025 Jun 14;8:928. doi: 10.1038/s42003-025-08357-5 (PMC12167378; doi:10.1038/s42003-025-08357-5)
Supplement: Supplementary file 5 — Reporting summary [file 42003_2025_8357_MOESM5_ESM.pdf]

nature portfolio

Corresponding author(s): Jae-Byum Chang and Young-Gyu Yoon

Last updated by author(s): Jun 3, 2025

Reporting Summary

Nature Portfolio wishes to improve the reproducibility of the work that we publish. This form provides structure for consistency and transparency in reporting. For further information on Nature Portfolio policies, see our Editorial Policies and the Editorial Policy Checklist.

Statistics

For all statistical analyses, confirm that the following items are present in the figure legend, table legend, main text, or Methods section.

n/a	Confirmed	
☐	☒	The exact sample size (*n*) for each experimental group/condition, given as a discrete number and unit of measurement
☐	☒	A statement on whether measurements were taken from distinct samples or whether the same sample was measured repeatedly
☐	☒	The statistical test(s) used AND whether they are one- or two-sided<br>*Only common tests should be described solely by name; describe more complex techniques in the Methods section.*
☒	☐	A description of all covariates tested
☒	☐	A description of any assumptions or corrections, such as tests of normality and adjustment for multiple comparisons
☒	☐	A full description of the statistical parameters including central tendency (e.g. means) or other basic estimates (e.g. regression coefficient) AND variation (e.g. standard deviation) or associated estimates of uncertainty (e.g. confidence intervals)
☐	☒	For null hypothesis testing, the test statistic (e.g. *F*, *t*, *r*) with confidence intervals, effect sizes, degrees of freedom and *P* value noted<br>*Give P values as exact values whenever suitable.*
☒	☐	For Bayesian analysis, information on the choice of priors and Markov chain Monte Carlo settings
☒	☐	For hierarchical and complex designs, identification of the appropriate level for tests and full reporting of outcomes
☐	☒	Estimates of effect sizes (e.g. Cohen's *d*, Pearson's *r*), indicating how they were calculated

Our web collection on statistics for biologists contains articles on many of the points above.

Software and code

Policy information about availability of computer code

Data collection	Fusion v2.3.0.44 (for Dragonfly Andor spinning disk confocal)
Data analysis	ImageJ/Fiji v1.53f51, MATLAB R2023a v9.14, Python v3.9

For manuscripts utilizing custom algorithms or software that are central to the research but not yet described in published literature, software must be made available to editors and reviewers. We strongly encourage code deposition in a community repository (e.g. GitHub). See the Nature Portfolio guidelines for submitting code & software for further information.

Data

Policy information about availability of data

All manuscripts must include a data availability statement. This statement should provide the following information, where applicable:

- Accession codes, unique identifiers, or web links for publicly available datasets
- A description of any restrictions on data availability
- For clinical datasets or third party data, please ensure that the statement adheres to our policy

The data generated in this study are available from the corresponding authors upon reasonable request. The public human cell dataset from Allen Institute for Cell Science can be downloaded from https://downloads.allencell.org/publication-data/label-free-prediction/index.html.

Research involving human participants, their data, or biological material

Policy information about studies with human participants or human data. See also policy information about sex, gender (identity/presentation), and sexual orientation and race, ethnicity and racism.

Reporting on sex and gender	n/a
Reporting on race, ethnicity, or other socially relevant groupings	n/a
Population characteristics	n/a
Recruitment	n/a
Ethics oversight	n/a

Note that full information on the approval of the study protocol must also be provided in the manuscript.

Field-specific reporting

Please select the one below that is the best fit for your research. If you are not sure, read the appropriate sections before making your selection.

☒ Life sciences ☐ Behavioural & social sciences ☐ Ecological, evolutionary & environmental sciences

For a reference copy of the document with all sections, see nature.com/documents/nr-reporting-summary-flat.pdf

Life sciences study design

All studies must disclose on these points even when the disclosure is negative.

Sample size	Each dataset presented in the manuscript corresponds to a single z-stack image, consisting of multiple z slices.<br>For mouse brain images, we utilized each z-stack image of 1024 (x) × 1024 (y) × 26 (z) pixels, corresponding to a volume of 204.8 µm × 204.8 µm × 25 µm. The feature extraction network dataset consists of four z-stack images for each protein, acquired from different fields of view within the same anatomical region from one animal. We utilized three z-stacks for each protein as training data and one z-stack for test data to calculate feature-based distances and identify the optimal grouping. The protein separation network dataset includes four z-stack images for each protein pair, acquired from different fields of view within the same anatomical region from two independent animals. For each protein pair, three z-stacks were used to train the protein separation network, and one z-stack was used to evaluate the unmixing performance and analysis the relationship between the unmixing performance and the feature-based distance.<br>For publicly available human cell images, we utilized each z-stack image of 924 (x) × 624 (y) × 24 (z) pixels, with a pixel size of 0.108 µm and inter z-slice interval of 0.29 µm. For the feature extraction network, we used 40 z-stacks for each protein to train the network and 11 z-stacks for each protein to extract the feature vector and identify the protein pairs. For the protein separation network, we used 40 z-stacks for each pair of two proteins to train the network and 11 z-stacks for each pair of two proteins to evaluate the unmixing performance.
Data exclusions	Due to the deduction in antibody concentration with staining depth, data from regions where few signal was detected were excluded.
Replication	We conducted cross-validation analysis using four independent datasets to demonstrate reproducibility, and detailed analysis of how imaging conditions affect performance, including resolution effects and noise impact. Also, we validate our method across more samples(n=50) on different tissues/antigens (human cell dataset).
Randomization	We used randomly selected 3 samples among 4 acquired images for training.
Blinding	Not applicable, as there were no such experimental groups in this study.

Reporting for specific materials, systems and methods

We require information from authors about some types of materials, experimental systems and methods used in many studies. Here, indicate whether each material, system or method listed is relevant to your study. If you are not sure if a list item applies to your research, read the appropriate section before selecting a response.

Materials & experimental systems

n/a	Involved in the study
☐	☒ Antibodies
☒	☐ Eukaryotic cell lines
☒	☐ Palaeontology and archaeology
☐	☒ Animals and other organisms
☒	☐ Clinical data
☒	☐ Dual use research of concern
☒	☐ Plants

Methods

n/a	Involved in the study
☒	☐ ChIP-seq
☒	☐ Flow cytometry
☒	☐ MRI-based neuroimaging

Antibodies

Antibodies used

1. Calbindin 2, ATLAS, HPA007305, Rabbit, Poly
2. Calnexin, Abcam, ab225995, Rabbit, Poly
3. Doublecortin, Abcam, ab18723, Rabbit, Poly
4. GFAP, ATLAS, HPA056030, Rabbit, Poly
5. Lamin B1, Abcam, ab16048, Rabbit, Poly
6. MAP2, Abcam, ab32454, Rabbit, Poly
7. NeuN, Millipore, ABN78, Rabbit, Poly
8. Nucleolin, Abcam, ab702, Rabbit, Poly
9. PV, Novus Bio, NB120-11427, Rabbit, Poly
10. S100B, Abcam, ab52642, Rabbit, Mono

Validation

Antibodies purchased from the Human Protein Atlas (HPA) were validated by the Human Protein Atlas project (Website: https://www.proteinatlas.org/), Detailed specifications and validation data for antibodies purchased from other vendors (Abcam, Novus Biologicals, Merck Millipore) can be found on the respective manufacturer's websites.

Animals and other research organisms

Policy information about studies involving animals; ARRIVE guidelines recommended for reporting animal research, and Sex and Gender in Research

Laboratory animals

C57BL/6J male mice ages 4-8 weeks were used for mouse brain imaging.

Wild animals

Not applicable.

Reporting on sex

Male mice were used.

Field-collected samples

Not applicable.

Ethics oversight

All the procedures involving animals were approved by the Korea Advanced Institute of Science and Technology Institutional Animal Care and Use Committee (KAIST-IACUC).

Note that full information on the approval of the study protocol must also be provided in the manuscript.

Plants

Seed stocks

n/a

Novel plant genotypes

n/a

Authentication

n/a
